# Stress-induced fetal programming contributes to the manifestation of Duchenne muscular dystrophy in *mdx* mice

**DOI:** 10.1016/j.isci.2025.112123

**Published:** 2025-03-01

**Authors:** Saba Gharibi, Gretel S. Major, Ali Shad, Bridgette D. Semple, Narelle E. McGregor, Martha Blank, Gavin Abbott, Natalie A. Sims, Christopher S. Shaw, Aaron P. Russell, Angus Lindsay

**Affiliations:** 1 School of Exercise and Nutrition Sciences, Institute for Physical Activity and Nutrition (IPAN), Deakin University, Geelong, VIC 3216, Australia; 2 School of Biological Sciences, University of Canterbury, Christchurch 8140, New Zealand; 3 Biomolecular Interaction Centre, School of Biological Sciences, University of Canterbury, Christchurch 8140, New Zealand; 4 Department of Neuroscience, The School of Translational Medicine, Monash University, Melbourne, VIC 3004, Australia; 5 Alfred Health, Prahran, VIC, Australia; 6 Bone Cell Biology and Disease Unit, St Vincent’s Institute of Medical Research, Fitzroy, VIC, Australia; 7 Department of Medicine, The University of Melbourne, St. Vincent’s Hospital, Melbourne, VIC, Australia; 8 Mary Mackillop Institute for Health Research, Australian Catholic University, Melbourne, VIC, Australia; 9 Department of Medicine, University of Otago, Christchurch 8014, New Zealand; 10 Maurice Wilkins Centre for Molecular Biodiscovery, Auckland 1010, New Zealand

**Keywords:** Natural sciences, Biological sciences, Physiology, Neuroscience

## Abstract

Prenatal stress predisposes offspring to neurocognitive, metabolic, and cardiovascular complications in adulthood, but the programming effects on the presentation of hereditary diseases are unknown. We investigate this in a mouse model of Duchenne muscular dystrophy (DMD), an X-linked neuromuscular disease that widely affects the central nervous system and peripheral tissues. Simulating the inheritance patterns of DMD by mating *mdx*-heterozygous females with wildtype males, we compare the central, autonomic, and peripheral phenotypes of healthy and DMD-affected (*mdx*) male offspring born from mothers that were either stressed or non-stressed during gestation. Prenatal stress predisposed *mdx* offspring to anxiety-like behavior and reduced bone mass but did not exacerbate stress hypersensitivity or skeletal muscle mass and function. In fact, prenatal stress increases blood pressure and may be protective against hypotension-induced mortality to stress. This demonstrates that offspring genetics influence the outcomes of fetal programming, and fetal programming influences the presentation of a hereditary disease.

## Highlights

• Prenatal stress impacts phenotypic outcomes in offspring born from DMD carriers
• *Mdx* mice from stressed mothers had improved hemodynamics
• Prenatal stress increased anxiety behavior and lowered bone mass in *mdx* offspring
• Muscle phenotypes were not affected by prenatal stress in *mdx* offspring

## Graphical abstract

**Figure:**
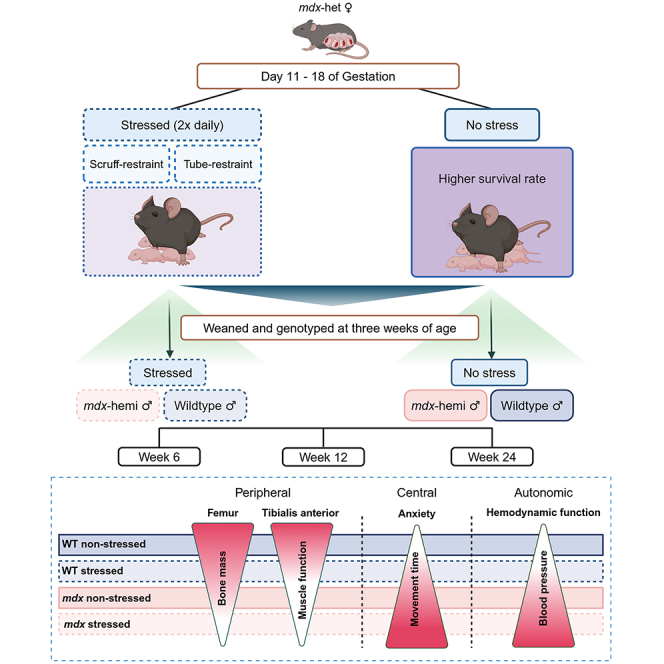

## Introduction

The Developmental Origins of Health and Disease theory highlights the critical contribution of the *in-utero* environment, or fetal programming, on the susceptibility to disease in adulthood.^1^ Primary evidence of this is the association between low body mass at birth and an increased risk of cardiovascular disease^2^^,^^3^^,^^4^^,^^5^^,^^6^ and type 2 diabetes^7^^,^^8^^,^^9^ later in life. Fetal malnutrition and fetal overexposure to glucocorticoids during critical periods of development cause structural, functional, and metabolic changes to the fetus, which have lifelong significance.^10^^,^^11^^,^^12^ These variable stress insults contribute to a highly plastic *in-utero* environment, which primes the fetus for specific environmental challenges before birth by altering organ structures, hormonal axes, the autonomic nervous system, and behavior.^12^

Hormonal programming can adjust the regulation and feedback systems of hormonal axes, including the hypothalamic-pituitary-adrenal (HPA) axis.^12^ Prenatal cortisol exposure and low birth weight can predict HPA axis function and reactivity later in life.^13^^,^^14^^,^^15^ In pre-clinical models, interventions that increase fetal exposure to glucocorticoids result in smaller offspring at birth, which then go on to develop components of metabolic syndrome and hyperactivation of the HPA axis in adulthood.^11^^,^^16^ Given the integration of HPA axis function and behavior, prenatal stress can also cause behavioral programming of offspring.^11^^,^^17^ In rodents, offspring born to prenatally stressed mothers have increased HPA hyperreactivity, decreased exploration, and increased freezing behaviors.^18^^,^^19^^,^^20^^,^^21^^,^^22^^,^^23^ In male offspring, this anxiety-like behavior and HPA axis dysregulation can be transmitted to future generations.^24^ Epigenetic mechanisms contribute to the development of behavioral and endocrine responses to stress in offspring by the behavioral transmission of brain gene expression patterns.^25^ Therefore, fetal programming may also contribute to the manifestation of stress-related disease in offspring postnatally.

Duchenne muscular dystrophy (DMD) is an X-linked genetic disease characterized by severe muscle wasting, metabolic dysfunction,^26^^,^^27^ and variable central nervous system dysregulation.^28^^,^^29^^,^^30^ DMD is caused by mutations in the gene encoding dystrophin, which in ∼75% of DMD cases is inherited recessively from carrier mother to son.^31^ Stress hypersensitivity is reported in patients with DMD and is replicated in mouse models of the disease.^32^^,^^33^^,^^34^^,^^35^^,^^36^^,^^37^ The *mdx* mouse model of DMD houses a point mutation in exon 23 of the dystrophin gene which causes a loss of the full-length dystrophin isoform (Dp427), however, the expression of the four shorter isoforms (Dp260, Dp140, Dp116 and Dp71) are still apparent through differential internal promotors within the gene.^38^ When challenged by stressful stimuli, the *mdx* mouse model of DMD exhibits stress-related tonic immobility, hypotension, and even cardiac failure.^32^^,^^33^^,^^34^^,^^35^^,^^39^ This heightened stress reactivity is a heterogeneous phenotype; however, repeated stressors can exacerbate muscular disease phenotypes in *mdx* mice.^33^^,^^34^ Because stress hypersensitivity is characteristic of DMD, fetal programming due to prenatal stress may predispose offspring to these manifestations and could contribute to an exacerbation of disease phenotypes.

While the ability of the *in-utero* environment to program the fetal central nervous system and neuroendocrine response is well established, the peripheral (musculoskeletal) consequences of prenatal stress on development are unclear. This is in part due to the complexity of longitudinal follow-up studies in humans but also due to limited focus on the peripheral tissues of offspring in preclinical models. It is also unclear whether prenatal stress can magnify phenotypes of genetically determined diseases. This would be valuable insight for understanding pathological heterogeneity in diseases such as DMD and mitigating stress-related phenotypes.

In this study, we report on the programming effects of prenatal stress on central, autonomic, and peripheral developmental outcomes in offspring born to DMD-carrier mothers and evaluate whether prenatal stress can prime the severity of genetically determined diseases. *Mdx*-heterozygous mothers were stressed twice daily during the last trimester of gestation, and the male offspring of two genotypes, wildtype (WT; dystrophin-positive) and *mdx* (dystrophin-negative)*,* were behaviorally and functionally profiled over a 24-week developmental period. A comprehensive evaluation of gestational outcomes, behavior, hemodynamic function, skeletal muscle, and bone development revealed that *mdx* offspring were more sensitive to prenatal stress programming than WT offspring. Prenatal stress led to an increase in preterm births and a reduction in the body mass of offspring of both genotypes. While stress hypersensitivity and muscle phenotypes were not affected by prenatal stress in *mdx* offspring, an increase in anxiety-like behavior and lowered bone mass was apparent. Interestingly, while *mdx* mice born to stressed mothers exhibited no change in the acute stress response, they had improved hemodynamic function during a stressor. These data provide evidence that prenatal stress programming influences the manifestation of some central, autonomic, and peripheral phenotypes in DMD and contributes to our understanding of the mechanisms driving stress hypersensitivity in DMD.

## Results

### Prenatal stress causes poor gestational outcomes in hereditary Duchenne muscular dystrophy carrier mothers

**Figure 1:**
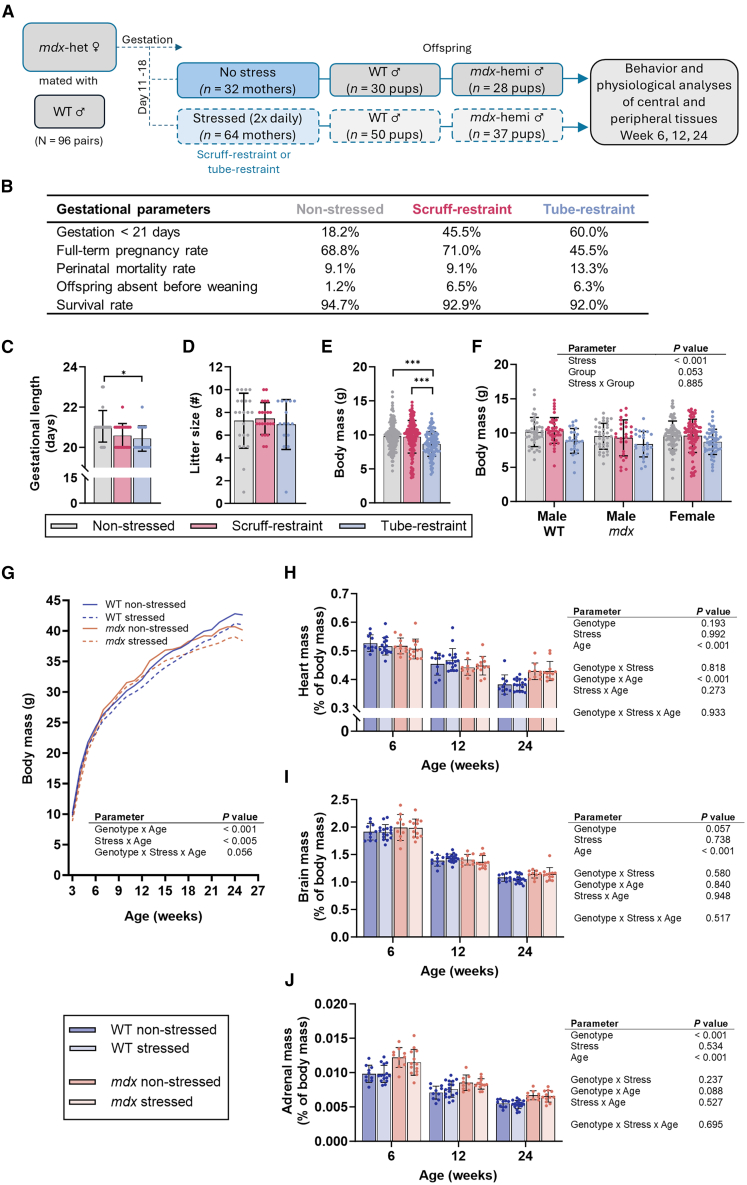
Stress during gestation affects gestational length, delivery success, and the size of offspring from C57BL/10-*mdx* heterozygous mothers (A) Experimental design. Ninety-six heterozygous female C57BL/10-*mdx* mice (*mdx*-het ♀) were paired with 96 C57BL/10 males (WT ♂) at 12–15 weeks of age. During the last week of gestation, pregnant *mdx*-het females were randomly assigned to one of the three groups: non-stressed, 30 s scruff-restraint twice daily, or 30 min of tube-restraint twice daily (*n* = 30/group). Male WT and *mdx*-hemi pups born to each stress paradigm were weaned and genotyped at three weeks of age, then divided into three age groups: 6, 12, and 24 for behavioral and physiological testing. (B) Gestation and birth statistics for offspring born to non-stressed, scruff-restrained, or tube-restrained female *mdx*-het mice. Full-term pregnancy rate; the percentage of mothers which were confirmed pregnant by the identification of vaginal plug and daily visual inspection and gave birth, Perinatal mortality; death of offspring immediately after birth, Offspring absent before weaning; the number of mice that failed to survive until weaning (3 weeks-of-age), Survival rate; the total number of offspring that were weaned (3 weeks-of-age) relative to the total number of offspring born. (C) Gestational length and (D) litter size of female *mdx*-het mice subjected to either non-stressed, scruff-restraint or tube-restraint paradigms during the last week of gestation (*n* = 16–22/group). (E) Body mass of all offspring (genotypes and sex) at three weeks of age (*n* = 100–155/group) and (F) body mass of offspring distinguished by sex and genotype at three weeks of age born to non-stressed, scruff-restrained, or tube-restrained female *mdx*-het mice (*n* = 27–83/group). Data were analyzed using a one or two-way ANOVA with Tukey’s post-hoc analysis. ∗*p* < 0.05, ∗∗∗*p* < 0.001. (G) Body mass of male wildtype (WT) and *mdx* offspring born to female *mdx*-het mice that during the last week of gestation were either non-stressed or stressed (either subjected to a 30 s scruff-restraint or a 30 min tube-restraint twice daily, data combined). Data were analyzed using a cubic polynomial model. *N* = 38–66/genotype/group. (H) Relative heart mass, (I) relative brain mass and (J) relative adrenal gland mass of male WT and *mdx* offspring born to female *mdx*-het mice that during the last week of gestation were either not stressed or stressed. Data were analyzed using three-way ANOVA. *N* = 10–17/genotype/group. All data are mean ± SD.

To mimic the recessive inheritance patterns of DMD in humans, *mdx*-heterozygous mothers (DMD carriers) were mated with WT males (Figure 1A). The nature and duration of prenatal stress, and the fetal developmental stage at which the stress occurs, affect the programming outcomes on the fetus.^40^ Stress in the third trimester is known to cause the greatest effects in neurocognitive and behavioral development because the fetus is particularly vulnerable to ischemia.^41^ Given that the cerebral cortex develops during late pregnancy, the motor system is also affected by late-stage gestational stress.^42^ On this basis, in the last week of gestation *mdx*-heterozygous mothers were either non-stressed or stressed twice daily by either scruff- or tube-restraint, and the behavioral and physiological development of the male WT and *mdx* offspring was evaluated in three separate cohorts over 24 weeks. To align with established prenatal stress paradigms, a 30 min tube restraint stress paradigm was employed.^20^^,^^43^^,^^44^^,^^45^ A 30 s scruff restraint paradigm was also selected because this stressor induces a robust acute stress response in the *mdx* mouse strain but has not yet been explored in the context of prenatal stress.^33^^,^^34^^,^^36^^,^^39^ By employing both these paradigms, we aimed to assess the effect of prenatal stress under a condition known to elicit significant physiological responses in *mdx* mice but also under a condition that aligns with the prenatal stress literature. To validate the stress paradigms used in this study, we evaluated corticosterone levels in *mdx*-heterozygous females acutely after scruff- and tube-restraint stressors (Figures S1A and S1B). There was a stress by time interaction over serum corticosterone, whereby both scruff- and tube-restraint stress paradigms caused a relative increase 30 min post stressor (*p* = 0.009) compared to the non-stressed paradigm (Figure S1B). No differences in serum corticosterone levels were identified between the scruff- and tube-restraint paradigms at any time point (Figure S1; *p* ≥ 0.514). However, corticosterone was elevated above pre-stressor levels 24 h after the tube-restraint stressor (Figure S1; *p* = 0.010). These results provide evidence that both the scruff and tube-restraint stress paradigms cause a similar acute stress response in *mdx*-heterozygous females, which appears more sustained following the tube-restraint.

Prenatal stress is known to affect gestation and birth characteristics, including the frequency of preterm births and fetal size at birth.^46^ Here, the prenatal stress paradigm differentially affected gestational and birth characteristics, where tube-restraint stress had a greater effect than scruff-restraint stress (Figures 1B and 1C). Gestational length was lower in tube-restrained mothers relative to non-stressed mothers (*p* = 0.018), with a ∼42% increase in pre-term births, ∼23% decrease in full-term pregnancies, and a ∼3% increase in perinatal mortality (Figures 1B and 1C). The stress paradigms employed did not affect litter size (*p* = 0.745), but the proportion of offspring that died prior to weaning was increased in both stress paradigms relative to the non-stressed paradigm (Figures 1B and 1D). There was evidence of increased offspring grooming at weaning in response to prenatal stress. Among the offspring of scruff-restrained and tube-restrained mothers, 60% and 65%, respectively, were classified as excessively groomed. In contrast, 5% of offspring born to non-stressed mothers were identified as excessively groomed at weaning. Prenatal tube-restraint stress resulted in offspring with lower body mass relative to offspring born to non-stressed and scruff-restraint stressed mothers (Figure 1E; *p* < 0.001). The lower birth weight with prenatal stress was independent of the offspring genotype or sex (Figure 1F; interaction *p* = 0.885). These data align with previous reports of prenatal stress on gestational outcomes,^46^ validating the approach taken. Despite differences in gestational outcomes, very few differences were identified between scruff- or tube-restraint stress paradigms in all offspring developmental outcomes (Tables S1–S8). Therefore, both stress paradigms were combined for all further analyses.

### Prenatal stress lowers body mass but does not affect organ development

As DMD is a recessive disease that predominantly affects males, only male offspring were investigated longitudinally to determine if there was an association between prenatal stress and the manifestation of disease phenotypes. There was an interaction between offspring genotype and age over body mass with aging (*p* < 0.001), as well as prenatal stress and age (Figure 1G; *p* < 0.005). Consistently with age and independent of genotype, offspring born to stressed mothers were smaller than offspring born to non-stressed mothers (Figure 1G). There were no effects of prenatal stress on the mass of the heart, brain, or adrenal gland at any age assessed (Figures 1H–1J; *p* ≥ 0.534). There was a genotype by time interaction over heart mass relative to body mass, whereby at 24 weeks of age, the hearts of *mdx* offspring were 14.8% greater in mass compared with WT offspring (Figure 1H; Table S9; *p* < 0.001). Irrespective of age, the adrenal glands of *mdx* offspring had a greater mass relative to body mass compared to those from WT offspring (Figure 1J; Table S9; *p* < 0.001). These data present clear genotypic differences in adrenal gland size from early development and indicate that the lower body mass observed throughout development in offspring born from stressed mothers, regardless of genotype, does not result in smaller vital organs.

### Anxiety in *mdx* offspring is intensified by prenatal stress, whereas wildtype offspring are unaffected

**Figure 2:**
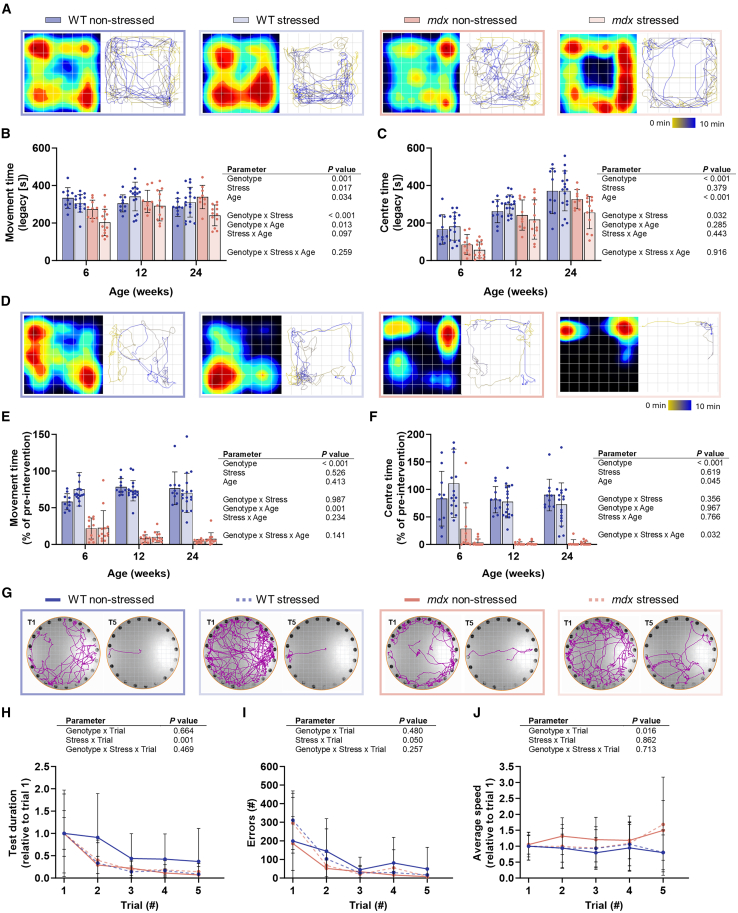
Gestational stress causes increased anxiety in offspring born to C57BL/10-*mdx* heterozygous mothers in a genotype-specific manner Behavioral profiling of male wildtype (WT) and *mdx* mice born to female *mdx*-het mice that during the last week of gestation were either not stressed or stressed. (A–C) Exploratory open field physical activity and (D-F) open field physical activity after an acute 30 s scruff-restraint stressor (data presented as the ratio of post-stressor physical activity to pre-stressor physical activity (% of intervention)). (A), (D) Representative heatmaps and movement traces at 6 weeks of age. In heat maps, red indicates more time spent in that region; blue indicates less time spent in that region. In movement traces, yellow indicates the mouse location early in the test; blue indicates the mouse location late in the test. (B), (E) Duration of activity and (C), (F) time spent in the center portion of the open field activity monitoring cage. Data were analyzed using three-way ANOVA. *N* = 10–17/genotype/group. (G–J) Memory of 24-week-old offspring in Barnes maze. (G) Representative locomotion traces of trial number one and five of offspring at 24 weeks of age. (H) Duration of the test, (I) total number of errors encountered in locating the escape hole and (J) the average speed of mice within the zone. Data were analyzed using the cubic polynomial model. *N* = 10–17/genotype/group. All data are mean ± SD.

It is hypothesized that prenatal stress is a developmental risk factor for cognitive and psychosocial vulnerabilities in offspring.^47^^,^^48^^,^^49^^,^^50^^,^^51^ Emotional and intellectual comorbidities affect up to 44% of patients with DMD^52^ and have been associated with worse prognostic outcomes.^53^ We therefore investigated whether prenatal stress contributes to this phenotype in DMD by longitudinally profiling offspring behavior (anxiety, stress, and memory testing). There was a genotype effect on most anxiety-like and stress parameters evaluated (Figures 2B, 2C, 2E, 2F, and S2C–S2F; Tables S10 and S11; *p* ≤ 0.026). Open-field activity monitoring was used to measure anxiety-like behavior in a novel environment (Figures 2A–2C and S2A–S2C). *Mdx* offspring were more greatly impacted by prenatal stress than WT offspring in the open field (Figures 2A–2C). There was an interaction between genotype and prenatal stress on movement time (*p* < 0.001), as well as between genotype and age (Figure 2A; *p* < 0.013). Across all ages, *mdx* offspring born to stressed mothers spent ∼21% less time moving in the novel environment relative to *mdx* offspring born to unstressed mothers (Figure 2A; Table S10). There was an interaction between genotype and stress on time spent in the center of the open field (*p* = 0.032), as well as an effect of age independent of stress or genotype (Figure 2B; Table S10; *p* < 0.001). At 6 weeks of age, *mdx* offspring born to stressed and non-stressed mothers spent less time in the center of the open field relative to WT offspring (Figure 2B; Table S10). With increasing age, offspring from both WT and *mdx* genotypes increased their time spent in the center of the open field by ∼53% and ∼76%, respectively (Figure 2B; Table S10). Nevertheless, these data indicate that prenatal stress increases anxiety-like behaviors in *mdx* offspring but has no effect on anxiety in WT offspring.

The acute stress response was assessed by evaluating open-field movement after a 30-s scruff-restraint (Figures 2D–2F and S2D–S2F), which causes an acute increase in serum corticosterone and sustained periods of physical inactivity in *mdx* mice.^34^ Prenatal stress had no effect on the acute stress response in either offspring genotype at any age assessed (Figures 2D–2F and S2D–S2F; Table S11; *p* ≥ 0.104). *Mdx* offspring were more affected by acute stress than WT offspring (Figures 2D–2F; Table S11; *p* ≤ 0.001). There was an interaction between genotype, stress, and age over time spent in the center of the open field and total distance ambulated (Figures 2E and S2D; Table S11; *p* = 0.032). These data indicate that prenatal stress does not affect the acute stress response in the offspring of either genotype.

Next, we assessed memory in the Barnes maze over repeated trials (Figures 2G–2J and S3). At all ages assessed, test duration and errors decreased over consecutive trials, suggesting memory retention in all groups (Figures 2H, 2I, S3A, S3D, and S3G). There was no effect of prenatal stress on memory in WT or *mdx* offspring at 6 and 12 weeks of age (Figure S3), indicating that both *mdx* and WT offspring demonstrated comparable memory at these times (Figure S3; *p* ≥ 0.096). At 24 weeks, there was a stress by trial interaction over test duration and number of errors made in identifying and entering the escape hole (Figures 2H and 2I; *p* ≤ 0.050). WT offspring born to non-stressed mothers completed the test more slowly, spent less time moving, and improved less over repeated trials compared with all other offspring (Figures 2H, 2I, and S3H). There was also a genotype by trial effect over the average speed of the offspring during the test (Figure 2I; *p* = 0.016). By trial five, *mdx* offspring had a ∼2.7-times greater relative increase in speed compared to that of WT offspring (Figure 2I). These data suggest that prenatal stress increases memory capacity in older WT offspring but had no effect on *mdx* offspring.

### Prenatal stress normalizes the weakened hemodynamic response to stress in *mdx* offspring

**Figure 3:**
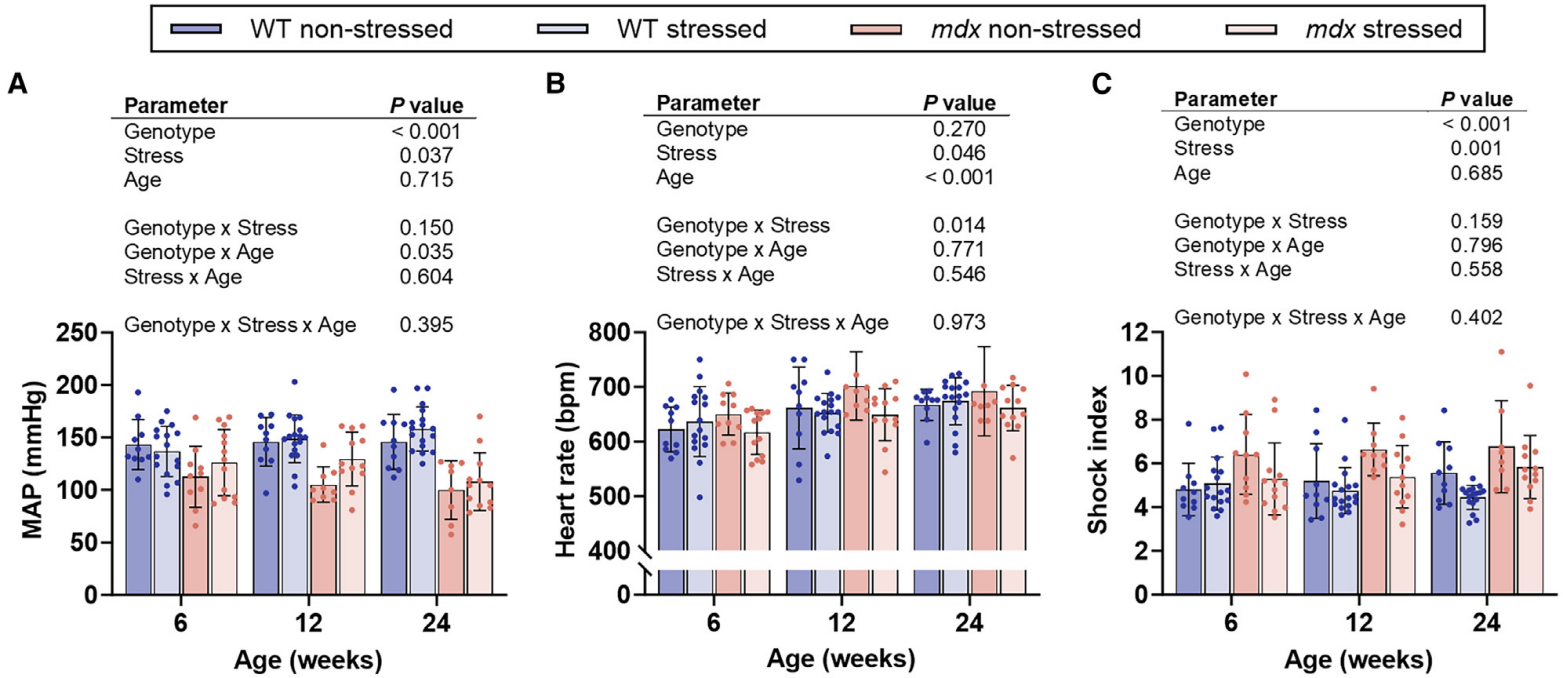
Gestational stress in C57BL/10-*mdx* heterozygous mothers improves the hemodynamic response of mdx offspring to an acute tube-restraint stress (A) Mean arterial pressure (MAP), (B) heart rate and (C) shock index (maximum heart rate/low systolic blood pressure; index of hypovolemic shock) of male wildtype (WT) and *mdx* mice born to female *mdx*-het mice that during the last week of gestation were either not stressed or stressed. Data were analyzed using a three-way ANOVA or cubic polynomial model. *N* = 10–17/genotype/group. All data are mean ± SD.

The dysfunctional stress response in the *mdx* mouse model of DMD manifests as acute, and sometimes sustained, hypotension and an increased shock index in response to stress, which can result in mortality when exposed to severe stress paradigms.^32^^,^^35^ To assess whether the dysfunctional hemodynamic response of *mdx* mice is affected by prenatal stress, the hemodynamic response of offspring was monitored throughout a 5-min tube-restraint stressor. There was no difference in median mean arterial pressure (MAP), shock index, or heart rate in WT offspring born to non-stressed or stressed mothers (Figures 3 and S4). However, there was an effect of genotype (*p* < 0.001) and prenatal stress (*p* ≤ 0.037) on median MAP and shock index (Figures 3A, 3B, and S4; Table S12). A genotype by age effect was identified over MAP (*p* = 0.035), whereby at 12 and 24 weeks *mdx* mice born to non-stressed mothers had a ∼0.7-fold lower median MAP relative to WT offspring born to both non-stressed and stressed mothers (Figures 3A and S4; Table S12). At 6 and 12 weeks of age, the MAP of *mdx* offspring born to stressed mothers was 13 and 25 mmHg higher than *mdx* offspring born to non-stressed mothers (Figure 3; Table S12). In *mdx* offspring, those born to stressed mothers had a ∼0.8-fold lower shock index relative to those born to non-stressed mothers (Figure 3B). There was an effect of age (*p* < 0.001) and prenatal stress (*p* = 0.046) on heart rate, as well as a genotype by stress interaction (Figure 3C; Table S12; *p* = 0.014). The heart rate of WT offspring born to non-stressed and stressed mothers did not differ (Figure 3C; Table S12). *Mdx* offspring born to stressed mothers had a ∼6% reduction in heart rate relative to *mdx* offspring born to non-stressed mothers (Figure 3; Table S12). These data indicate that prenatal stress can improve the hemodynamic response to acute stress in *mdx* offspring by increasing MAP and decreasing heart rate to levels similar to WT offspring.

### Muscle development and function are unaffected by prenatal stress independent of offspring genotype

**Figure 4:**
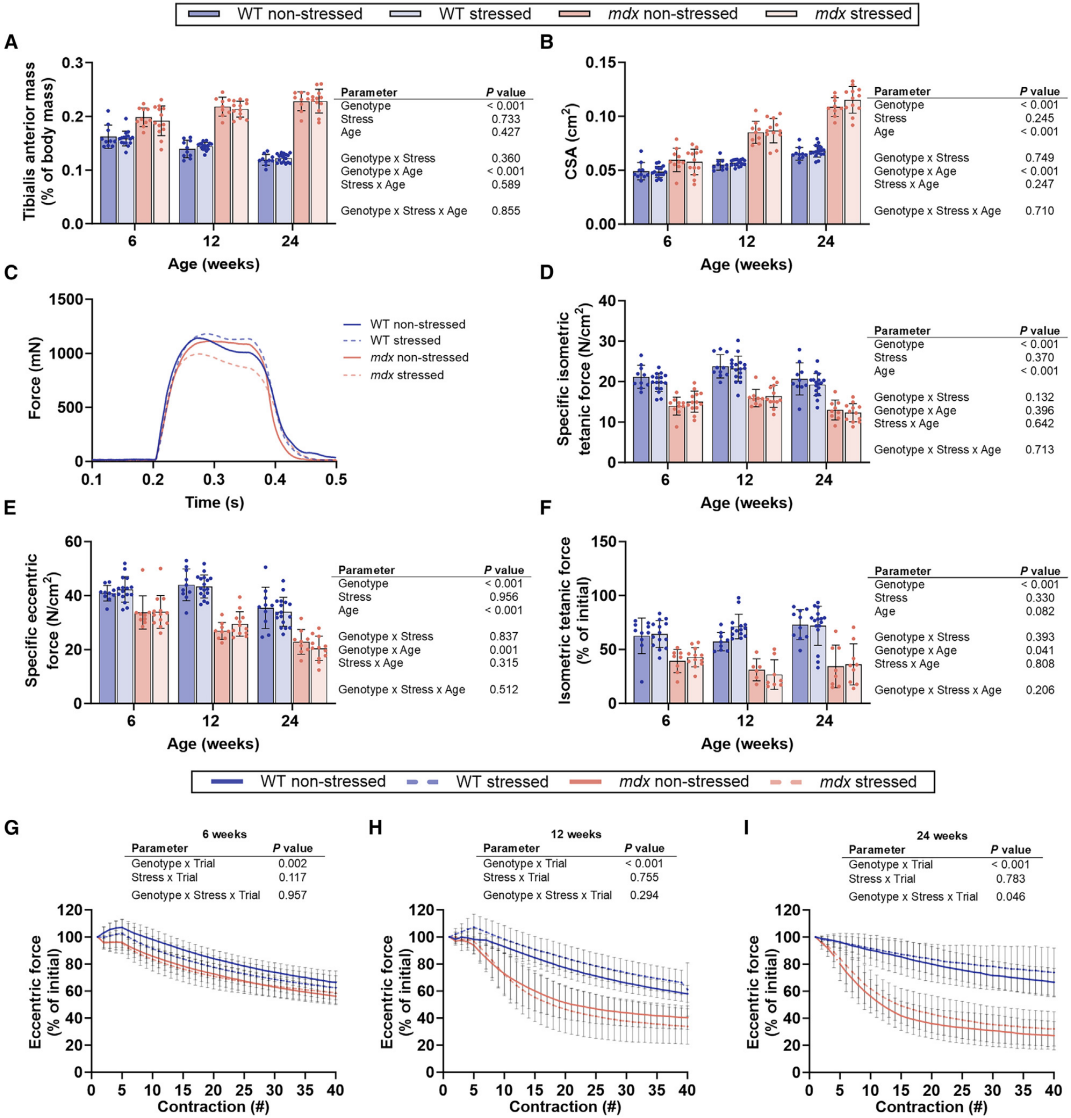
Muscle mass, force production, eccentric strength, and sensitivity to muscle damage from eccentric contraction are not affected by gestational stress in offspring from C57BL/10-*mdx* heterozygous mothers Analysis of the tibialis anterior from male wildtype (WT) and *mdx* mice born to female *mdx*-het mice that during the last week of gestation were either not stressed or stressed. (A) Relative muscle mass, (B) cross-sectional area (CSA), (C) representative tetanic force curves at 12 weeks of age, (D) specific isometric tetanic force, (E) specific eccentric force, (F) the percentage of isometric force lost following eccentric contractions, and (G), (H), (I) loss of eccentric force at 6, 12 and 24 weeks of age, respectively. Data were analyzed using a three-way ANOVA or cubic polynomial model. *N* = 6–17/genotype/group. All data are mean ± SD.

The predominant manifestation of DMD is severe and progressive muscle damage and loss of function.^54^ It is unknown how prenatal stress affects the development of peripheral tissues, such as muscle, and whether the *in-utero* environment can contribute to the severity and progression of genetic disease phenotypes. Consequently, muscle mass, strength, and sensitivity to contraction-induced damage were evaluated with age. There was no effect of prenatal stress on the mass of the tibialis anterior, gastrocnemius, or soleus muscles (normalized to body mass) (Figure 4A; Tables S13 and S14; *p* ≥ 0.399). The tibialis anterior of the *mdx* offspring had a greater mass and cross-sectional area relative to WT offspring (Figures 4A and 4B; Tables S13 and S14; ∼17%, ∼33% and ∼33% larger cross-sectional area at 6, 12 and 24 weeks, respectively; *p* < 0.001). The cross-sectional area of the tibialis anterior from *mdx* offspring increased ∼1.8-fold from 6 weeks to 24 weeks of age (Figure 4B; Table S14; *p* < 0.001).

Prenatal stress did not impact tibialis anterior muscle isometric or eccentric force production or contraction and relaxation rates *in situ* at any age assessed (Figures 4C–4E and S6; Table S14; *p* ≥ 0.232). WT offspring had a greater specific isometric tetanic force relative to *mdx* offspring at all ages assessed (Figure 4D; Table S14; *p* < 0.001). Specific isometric tetanic force was affected by the age of the offspring, whereby tetanic force was 13% higher between 6 and 12 weeks and ∼18% lower between 12 and 24 weeks (Figure 4D; Table S14; *p* < 0.001). There was a genotype by age effect for specific eccentric force and the proportion of isometric tetanic force lost following *in situ* eccentric contractions of the tibialis anterior (Figures 4E, 4F, and S6; Table S14; *p* ≤ 0.041). *Mdx* offspring demonstrated a ∼36% reduction in eccentric force between 6 and 24 weeks of age, which WT offspring did not replicate (Figure 4E). A genotype by trial effect was identified at all ages over the loss of eccentric strength with repeated contractions (Figures 4G–4I; *p* ≤ 0.002). At 24 weeks, there was a genotype by stress by trial effect identified over the loss of eccentric strength (Figures 4G–4I; *p* = 0.046). *Mdx* generally had a greater loss of eccentric strength with increasing contractions than WT offspring (Figures 4G–4I). Across all ages *mdx* offspring had lower force recovery relative to WT offspring acutely after eccentric contractions (Figures 4 and S6; Table S14). At 24 weeks of age, there was a genotype by stress by time interaction over the proportion of isometric tetanic force lost following eccentric contractions (Figures 4I and S6C; *p* = 0.046). This effect was not seen at 6 and 12 weeks of age (Figures 4H, S6A, and S6C; *p* ≥ 0.294). These data show that while the well-established loss of muscle force was observed in *mdx* mice with age, prenatal stress does not impact the size or function of the skeletal muscles of either offspring genotype.

### Early developmental deficits in bone structure in *mdx* offspring do not lead to bone fragility

**Figure 5:**
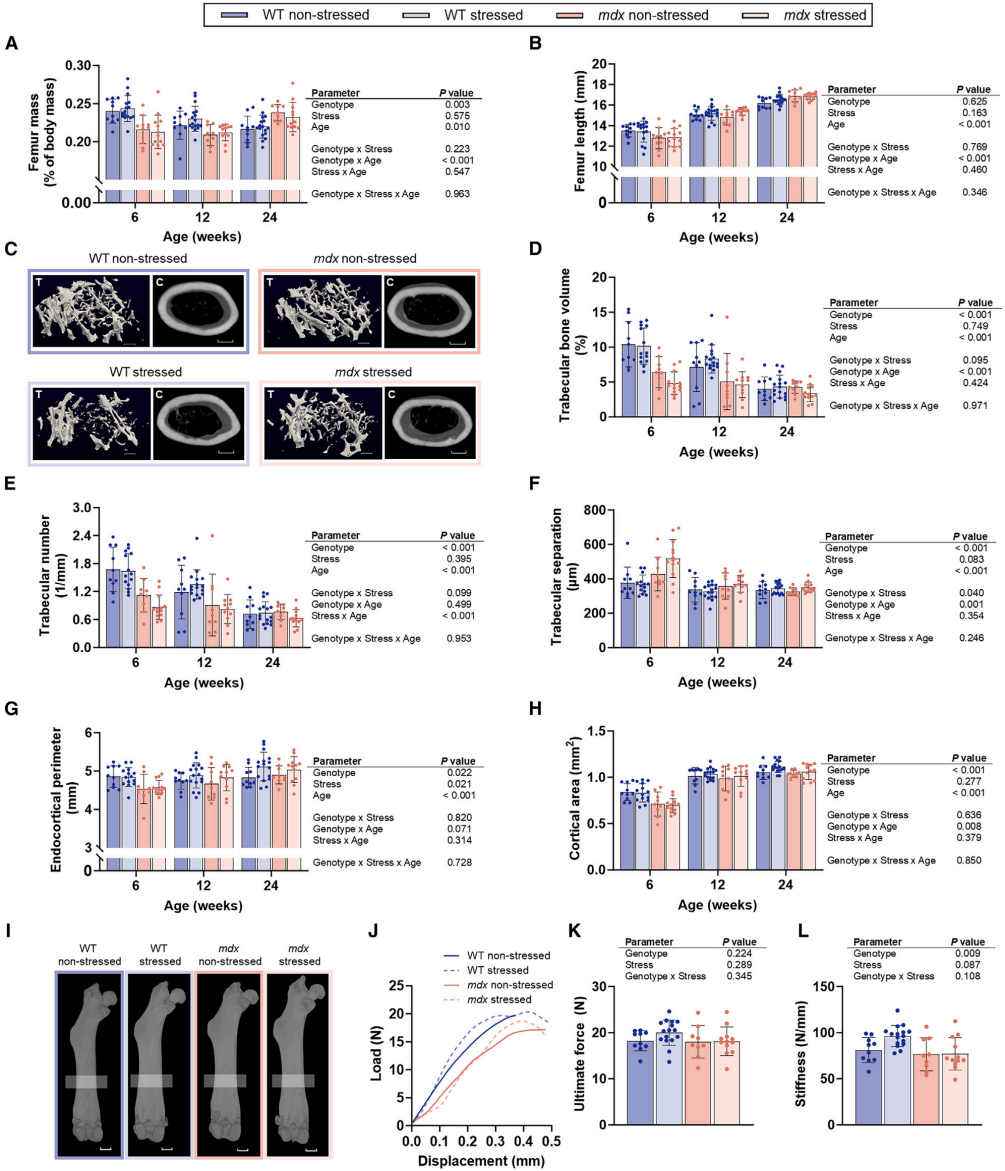
Stress during the last week of gestation impacts femoral trabecular structure in offspring born to C57BL/10-*mdx* heterozygous mothers Analysis of the femur from male wildtype (WT) and *mdx* mice born to female *mdx*-het mice that during the last week of gestation were either not stressed or stressed. (A) Relative femur mass, (B) femur length, and (C) representative micro-CT reconstructions of trabecular bone in the metaphysis and cortical bone in the diaphysis at 24 weeks of age. Scale bars are 0.2 mm and 0.5 mm for trabecular and cortical bone, respectively. (D) Trabecular bone volume (relative to tissue volume), (E) trabecular number, (F) trabecular separation, (G) endocortical perimeter, (H) cortical area, and (I) representative 3D reconstructions of the femur at 24 weeks of age (box indicates the cortical region of analysis). Scale bars are 1 mm. (J) Representative force-displacement curves at 24 weeks of age, (K) ultimate force and (L) bending stiffness of the femur in three-point bending tests of the mid-diaphysis. Data were analyzed using three-way ANOVA. *N* = 9–17/genotype/group. All data are mean ± SD.

As bone fragility is a manifestation of DMD^55^ and epidemiological studies have identified a link between low birth weight and osteoporosis in adulthood,^56^ the bone mass and structure of offspring were assessed. There was no effect of prenatal stress on the mass or length of the femur or tibia at any age assessed (Figures 5A, 5B, and 5I; Table S15; *p* ≥ 0.163). The effect of genotype on femur mass (normalized to body mass), femur length, trabecular bone volume, trabecular separation, endocortical perimeter and cortical bone area differed with age, being most notably altered at 6 weeks, and recovered by 24 weeks of age (Figures 5A, 5B, 5D, and 5F–5H; Table S15; *p* ≤ 0.071). At 6 weeks, *mdx* offspring born to stressed mothers had a 1.2-fold greater trabecular separation, indicating lower trabecular bone mass, relative to *mdx* offspring born to non-stressed mothers (genotype by stress interaction *p* = 0.040), although this was not statistically significant for trabecular bone volume (*p* = 0.095) or trabecular number (*p* = 0.099) (Figures 5D–5F; Table S15). There was a stress by age effect on trabecular number (*p* < 0.001) and a genotype by age effect on trabecular separation (Figures 5E and 5F; Table S15; *p* = 0.001). Across all trabecular parameters, the greatest genotype and stress-related differences in trabecular bone structure were identified at younger ages (6 and 12 weeks) (Figures 5D–5F; Table S15). In cortical bone, there was an effect of prenatal stress (*p* = 0.021) and genotype (*p* = 0.022) on endocortical perimeter, which was greater in the offspring of both genotypes born to stressed mothers at 12 and 24 weeks (Figure 5G; Table S15). There was no effect of stress on cortical bone area (*p* = 0.277), but there was a genotype by age effect (Figure 5H; Table S15; *p* < 0.008).

An increase in endocortical perimeter without an increase in cortical bone area was suggestive of compromised cortical structure in the femur. Therefore, to assess femur strength at the diaphysis a three-point bending test was conducted (Figures 5I–5L). Ultimate force was not significantly modified by prenatal stress or genotype (Figure 5K; *p* ≥ 0.224), and while there was also no effect of prenatal stress on bone stiffness (*p* ≥ 0.087), this parameter was modified by genotype (Figure 5L; Table S16; *p* = 0.009). Femora from WT offspring born from stressed mothers were stiffer than femora from *mdx* offspring (Figure 5L; Table S16; *p* = 0.009).

Overall, these data indicate that trabecular and cortical bone structure in *mdx* offspring are mildly compromised by prenatal stress but that these structural alterations do not result in reduced bone strength.

## Discussion

Prenatal stress can cause fetal programming of neurodevelopmental and endocrine axes, which predispose offspring to disease in adulthood. Here, we report on the capacity of prenatal stress in priming the severity of central, autonomic, and peripheral outcomes in DMD, a primarily hereditary disease. Prenatal stress in the last trimester resulted in adverse birth outcomes in healthy and dystrophin-deficient offspring. Prenatal stress had a greater effect on anxiety, the hemodynamic response to stress, and trabecular bone structure in *mdx* offspring relative to WT offspring. Importantly, prenatal stress did not exacerbate muscle weakness and contraction-induced damage, which are central pathological characteristics of DMD. These results identify a role for prenatal stress in programming the presentation of central (behavior), autonomic (hemodynamic function), and peripheral (osteopenia) phenotypes in DMD.

The incidence of adverse birth outcomes is linked with stress during critical windows of fetal development *in utero*. A predominant focus has previously been awarded to the effects of energy substrate availability on fetal outcomes; however, a reciprocal interaction between other maternal stressors and fetal nutrition is becoming clearer. For example, exposure to laboratory stressors and glucocorticoids can alter feeding behavior,^57^^,^^58^ restricting the growth and maturation of the fetus. We found here that tube-restraint stress in the last trimester caused an increased incidence of adverse birth outcomes, including reduced gestational length, increased perinatal and pre-weaning mortality rate, and low offspring body mass at weaning. Prenatal stress had a lasting effect on offspring body mass with age and did not predispose offspring to weight gain or obesity. This aligns with rodent studies demonstrating that molecular adipose tissue programming due to fetal overexposure to glucocorticoids does not result in adult obesity.^59^^,^^60^

Evolutionarily, preterm births may represent protective adaptations for both the mother and fetus to reduce stress exposure and preserve energy stores.^61^ However, premature delivery may pose additional costs to the fetus including increased perinatal and infant mortality, and rudimentary organ development.^61^ We found no effect of prenatal stress on the developmental size of the heart, brain, skeletal muscles or adrenal glands. Nevertheless, at 6 months of age *mdx* offspring had a greater heart mass relative to WT offspring, which is consistent with the cardiac hypertrophy and cardiomyopathy observed in DMD pathology.^62^^,^^63^ *Mdx* offspring also had larger adrenal glands relative to WT offspring throughout development, correlating with reports of *mdx* stress hypersensitivity and increased HPA axis activation.^33^ These longitudinal data provide evidence that *mdx* mice are internally wired for stress responsivity at early developmental stages irrespective of stressful *in-utero* conditions, and that this phenotype is not exacerbated as the disease progresses.

Circulating glucocorticoids are common hormones that convey information between mother and fetus during pregnancy. However, fetal overexposure to maternal glucocorticoids during neurodevelopment can adversely alter neural circuits that regulate behavior, learning, autonomic cardiac regulation, and blood pressure.^64^ Both WT and *mdx* offspring demonstrated reduced anxiety-like behaviors in the open field with increasing age, irrespective of prenatal stress. The reduced anxiety-like behaviors in *mdx* mice aligns with prior evidence in C57BL/6J mice that aging results in substantial alterations in the nervous system which reduce anxiety-like behaviors.^65^ We also observed that prenatal stress causes increased anxiety in *mdx* offspring but not WT offspring, highlighting a differential vulnerability to fetal programming between genotypes which has not been identified before. Furthermore, a similar increase in anxiety levels has only been demonstrated in more severe mouse models of DMD where both Dp427 and Dp140 dystrophin isoforms are missing, unlike the *mdx* mouse employed here which only lacks Dp427 (full-length dystrophin).^66^ From this we conclude that pre- and post-natal fetal programming is not a contributing mechanism for the stress hypersensitivity phenotype in DMD but may increase offspring susceptibility to anxiety-like behaviors. This is a significant finding in understanding emotional comorbidities in DMD, as anxiety disorders affect ∼24% of patients with DMD and are ∼3.7 times more prevalent than in healthy populations.^30^

We and others have reported on stress hypersensitivity in the *mdx* mouse model of DMD which causes the up-regulation of the HPA axis, tonic immobility, hypotension, increased hypovolemic shock, and in some cases cardiac damage.^32^^,^^35^^,^^36^^,^^37^^,^^39^ Prenatal stress is a well-known developmental risk factor for cardiovascular disease and can predispose offspring to hypertension in adulthood.^2^^,^^3^^,^^4^^,^^5^^,^^6^ We show that in *mdx* offspring prenatal stress caused an increase in median MAP, reduced hypovolemic shock, and normalized heart rate to WT levels in response to acute stress. This strengthens the link between prenatal stress and cardiovascular programming, which in *mdx* offspring seems capable of rescuing stress-induced hypotension. Given that prenatal stress did not salvage physical inactivity due to an acute stress, these data suggest that tonic immobility and the altered hemodynamic response to stress in *mdx* mice are discrete stress outcomes. While this cardiovascular programming appears persistent across ages, it is unclear whether it is protective of lethal phenotypes in response to severe subordinate stress.^35^

Muscle wasting causes a premature loss of ambulation and early fatality in DMD. Repetitive acute stress can accelerate muscle fibrosis in mature *mdx* mice^33^; however, here we report that prenatal stress does not have any impact on muscle mass, force production or sensitivity to contraction-induced damage in *mdx* or WT offspring. Compared with WT offspring, *mdx* offspring had larger skeletal muscle size and a reduced capacity to generate force, which became more extreme with age; phenotypes consistent with the increased muscle damage and fibrotic deposition characterizing disease manifestation in *mdx* mice.^67^^,^^68^^,^^69^ In *mdx* offspring, prenatal stress led to mild deficits in trabecular bone structure, and independent of offspring genotype, prenatal stress increased endocortical perimeter without increasing cortical area. While these effects did not reduce cortical bone strength, trabecular strength was not assessed. This would be worth investigating in further studies since trabecular bone mass is the main contributor to bone strength in vertebrae, and vertebral fractures are common in patients with DMD.^55^ The maternal environment is known to have long-lasting effects on offspring health, particularly relating to intrauterine calcium metabolism.^70^ Despite this, the effects of maternal stress on bone health are understudied with most work confined to assessing the effects of intra-uterine growth restrictions. This has detrimental effects on bone mass,^71^ largely due to its influence on birthweight, which may contribute to osteopenia throughout life.^56^ The current study provides further evidence for the fetal programming of peripheral tissues and suggests that bone structure is more sensitive to prenatal stress insults than skeletal muscle.

This study explores the effects of fetal programming on the phenotypic outcomes of DMD-affected and healthy offspring born to DMD carrier mothers. *Mdx* offspring were more sensitive to the effects of prenatal stress throughout development compared with WT offspring, but prenatal stress did not alter primary disease progression. The apparent vulnerability of *mdx* mice to stress suggests that the *in utero* environment, and not just genetics, affects the developmental outcomes of offspring. This is of critical importance as incidences of prenatal stress may contribute to the lower mortality rate reported in *mdx* cohorts and contribute to phenotypic variation between cohorts used for evaluating new therapies^72^ – therefore affecting future clinical trials for DMD. Researchers need to consider not just the impact of the therapy on the mouse itself but how the environment and animal husbandry may shape the outcomes of new therapies. Given that environmental stress experienced by mothers affects developmental outcomes, greater standardization of *mdx* breeding, maintenance and animal husbandry is necessary when generating new cohorts of *mdx* mice.^73^ To this end, suggestions for appropriate standard operating procedures have already been reported,^72^ however we suggest that greater transparency in reporting the breeding and research environment within articles is also necessary. This includes reporting other species held within research facilities (multiple or mouse only), number of personnel in contact or handling animals, frequency of cage disturbance and other noises and scents in the facility. Greater consideration of the prenatal environment may help reduce neonatal mortality and reduce potential variability across different cohorts of *mdx* mice or facilities.

In conclusion, by investigating DMD-affected and healthy offspring born to DMD carrier mothers, genotype-specific effects were identified, providing emphasis on the effect of offspring genetics on fetal programming outcomes. We highlight the central, autonomic, and peripheral phenotypes that are influenced by prenatal stress in healthy male offspring and in hereditary conditions such as DMD, reinstating the contribution of both genetic and *in-utero* environmental factors in the manifestation of disease. This is a critical line of questioning for other primarily hereditary diseases, which may support a greater need for maternal genetic screening and an increased role for genetic councilors in the education of the potential outcomes of maternal stress during pregnancy.

### Limitations of the study

A limitation of this work, given time constraints, was that a life-span study was not conducted, and hence, the consequences of prenatal stress on late-stage disease progression and survival rates could not be determined. Additionally, as DMD predominantly affects adolescent males, we focused on male offspring only. Given that prenatal stress is known to affect sexes differently,^74^ these findings have limited translation to healthy and carrier female offspring. Future studies should incorporate lifespan studies and consider both sexes to provide a true evaluation of the lifelong significance of prenatal stress on developmental outcomes in adulthood.

## Resource availability

### Lead contact

All data are available in the main text or the supplemental information. Illustrations have been created with biorender.com under a paid subscription by Deakin University. Further information and requests for resources and reagents should be directed to and will be fulfilled by the lead contact, Angus Lindsay (angus.lindsay@canterbury.ac.nz).

### Materials availability

This study did not generate new unique reagents.

### Data and code availability

• This study did not generate any large-scale sequencing or proteomics datasets.
• This article does not report original code or source code.
• Any additional information required to reanalyze the data reported in this article is available from the lead contact upon request.

## Acknowledgments

This work was supported by the Neurological Foundation (Philip Wrightson Fellowship, AL), 10.13039/501100001505Health Research Council of New Zealand (Sir Charles Hercus Health Research Fellowship; 23/037, AL), 10.13039/100015144Institute for Physical Activity and Nutrition (Seed Funding, AL) and 10.13039/501100001778Deakin University (Postgraduate Research Scholarship, SG). The authors would like to acknowledge the animal husbandry and care contributions of Claire Laird and Jennifer Davis.

## Author contributions

Conceptualization: AL, methodology: SG, AS, BDS, NEM, MB, GA, NAS, CSS, APR, and AL, investigation: SG, AS, BDS, NEM, MB, GA, NAS, and AL, visualization: SG and GSM, supervision: CSS, APR, and AL, writing—original draft: GSM, writing—review and editing: SG, AS, BDS, NEM, MB, GA, NAS, CSS, APR, and AL.

## Declaration of interests

All other authors declare they have no competing interests.

## STAR★Methods

### Key resources table

**Table:** 

REAGENT or RESOURCE	SOURCE	IDENTIFIER
**Chemicals, peptides, and recombinant proteins**		

Isoflurane Inhalation Anaesthetic	Pharmachem	CAS: 26675-46-7

**Critical commercial assays**		

Corticosterone ELISA	Arbor Assays	K014-HI

**Experimental models: Organisms/strains**		

C57BL/10ScSn^ARC^ males	Animal Resources Centre	N/A
C57BL/10ScSn^ARC^-*mdx* heterozygous females (female *mdx* heterozygous)	Animal Resources Centre	N/A
C57BL/10ScSn^ARC^-*mdx* hemizygous males	Generated in-house	N/A
C57BL/10ScSn^ARC^-*mdx* homozygous females	Generated in-house	N/A

**Software and algorithms**		

GraphPad Prism (version 9)	Graphpad	RRID:SCR_002798
ImageJ	NIH	RRID:SCR_003070
Stata/SE 17	StataCorp	RRID:SCR_012763
Fiji	NIH	RRID:SCR_002285
CODA® Data Acquisition Software	Kent Scientific	RRID:SCR_018585
Dynamic Muscle Control and Analysis Software	Aurora Scientific	https://aurorascientific.com/
NRecon	Bruker	https://www.bruker.com/
Dataviewer	Bruker	RRID:SCR_002633
CTAn	Bruker	https://www.bruker.com/
CTVox	Bruker	https://www.bruker.com/
ParaReview	Bruker	https://www.bruker.com/

### Experimental model and study participant details

#### Study approval

All studies received approval from the Deakin University Animal Ethics Committee (G04-2020). The housing and treatment of all animals adhered to the standards established by the Deakin University Animal Welfare Committee, complying with the ethical principles outlined in the Australian Code for the Care and Use of Animals for Scientific Purposes. This study is reported in accordance with the ARRIVE guidelines.

Our study exclusively examined male offspring because DMD predominantly affects males. All mice were on the C57BL/10ScSn^ARC^ background and were acquired from Animal Resources Centre (ARC) in Perth, Western Australia: C57BL/10ScSn^ARC^ males (wildtype; WT), C57BL/10ScSn^ARC^-*mdx* hemizygous males (male *mdx*), C57BL/10ScSn^ARC^-*mdx* homozygous females (female *mdx*), and C57BL/10ScSn^ARC^-*mdx* heterozygous females (female *mdx* heterozygous). All animals were housed in groups of four per cage. The room was maintained on a 12/12 h light/dark cycle with humidity between 40 - 70% and temperature at 21 ± 2°C. Food and water were provided *ad libitum* and the welfare of the animals were monitored daily.

#### Experimental design

At 12-15 weeks of age, 120 female *mdx* heterozygous mice were randomly paired with 120 male WT mice. The presence of a vaginal plug was designated as day 1 of gestation and these were checked twice/daily. Male mice were removed from the cage on gestational day 11. If no vaginal plug was detected, the male mice remained with the female for two weeks. Females that did not become pregnant on the first attempt were paired again with a male that had previously succeeded in breeding with another female. Then, pregnant female *mdx* heterozygous mice were randomly assigned to one of three groups: unstressed, 30 s scruff-restraint, or 30 min of tube-restraint (n = 31 – 33/group), as described below. After each parturition, gestation length and litter size were recorded. Pups were weaned and weighed at three weeks of age and ear punches from male mice were genotyped (Transnetyx, Cordova, TN, USA). Instances of cannibalism, macrocephaly, cataracts and pups defined as runts (stunted, undersized, or smaller relative to their littermates even after supplemental feeding with crushed or powdered moistened food and water in the cages) were recorded and excluded from further study. All remaining male WT and *mdx* pups born from each stress paradigm were then randomly allocated into three age groups: 6, 12, and 24 weeks (n = 6 – 10/stress paradigm/time-point). Mice allocated to each time point completed behavioral and physiological assessments to evaluate anxiety, stress, cardiovascular function, memory, and skeletal muscle function. The three separate cohorts, representing each time point, ensured that all mice were naïve to all behavioural and physiological testing. While still under anesthesia following skeletal muscle function testing, mice were cervically dislocated, and tissues dissected, weighed and frozen in liquid nitrogen-cooled isopentane and stored at −80°C until analysis. After death, femora were removed and frozen in dry ice for later analysis of bone morphology.

### Method details

#### Corticosterone

Blood (30 μL in a heparinized capillary tube) from the tail vein was collected under isoflurane anesthesia (1 - 3% isoflurane) from female *mdx* heterozygous mice (13 – 15 weeks of age; n = 7 – 8/group) 24 h prior to either no stress, 30 s scruff-restraint or 30 min tube-restraint. Blood was also collected 30 minutes, 3 h and 24 h after the intervention, with selected time points based on established corticosterone response patterns in *mdx* mice.^35^ Blood was allowed to clot at room temperature for 30 min, before being spun at 20,800 g for 10 min. Serum was removed and stored at -80°C in a clean tube until analysis. Corticosterone concentration was quantified using a Corticosterone Enzyme Immunoassay Kit (ELISA) following the manufacturer's instructions (Arbor Assays: K014-HI, Ann Arbor, MI, USA).

#### Scruff restraint

The mouse was restrained by the nape between the index finger and thumb, while securing the tail between the fourth and fifth fingers.^34^^,^^35^^,^^75^ The mouse was then oriented into the supine position for 30 seconds.

#### Tube-restraint

The mouse voluntarily entered a 3.5 cm diameter tube for 30 min, which allowed ample ventilation but did not enable the mice to turn around.^20^^,^^39^

#### Anxiety

Mice were placed in an open-field activity monitoring chamber for 10 min, with total distance ambulated (m), vertical activity (rearing), movement time (s), and time spent in the corner, center, or side of the chamber measured (s).^34^^,^^76^ These metrics were quantified using vertical and horizontal beam breaks recorded by the apparatus (AccuScan, Omnitech Electronics, Inc., USA). Chambers were sanitized with 70% ethanol between each session.

#### Stress

Mice underwent a 30 s scruff-restraint to initiate stress,^34^ and immediately placed in a cleaned and sterilized open-field activity monitoring chamber. Physical activity (total distance ambulated [m], vertical activity [rearing], movement time [s], and time spent in the corner, center, or side of the chamber measured [s] was measured for 10 min and compared to the level of activity completed in the anxiety test.

#### Stress hemodynamics

Mice were placed in the CODA® non-invasive blood pressure system using a 25 – 50 g mouse holder (Kent Scientific, Torrington, CT, USA) for 5 min.^77^ Customized tube restraints immobilized each mouse according to size, ensuring minimal movement. The setup included a 37°C warming platform, with a tail cuff (suitable for mice weighing 8 – 75 g) positioned to expose only the tip of the tail for consistent measurements across cohorts. Blood pressure was automatically recorded every 30 s for a total of 10 measurements using volume pressure recording (VPR) sensor technology and CODA software. Mean arterial pressure, heart rate and shock index (the ratio of maximum heart rate to lowest systolic blood pressure, indicating hypovolemic stress) were recorded.

#### Memory

Short-term memory was assessed by evaluating performance in a Barnes maze over repeated trials (Ugo Basile, Gemonio, VA, Italy). One hole was designed as a safety or escape hole, which remained unchanged throughout five trials. Mice were placed in the centre of the maze for five trials, 30 min apart. Similar shortened protocols have shown efficacy in distinguishing short-term spatial memory deficits in rodents^78^^,^^79^ and in this case was chosen to minimise external variability and to account for the experimental timeline. Test duration (s), average speed (m/s), maximum speed (m/s), errors (count) and total distance travelled (m) by mice to find the safety hole was captured and recorded using a video camera above the center of the maze (ANY maze, Stoelting Co, USA). The maze and escape were cleaned with 70% ethanol between each trial.

#### Skeletal muscle function

Mice were maintained under 1 – 3% isoflurane anaesthesia on a 37°C warming platform. After removing the skin and fascia, the distal tendon of the tibialis anterior (TA) was attached to the lever arm of a dual-mode servomotor using 5-0 silk suture (Aurora Scientific Inc. 305C-LR: Dual-Mode Footplate, Aurora, ON, Canada) and electrodes placed 3 mm apart under the sciatic nerve. The knee was securely attached to the apparatus (Aurora Scientific Inc. 809C: *in-situ* mouse apparatus). The sciatic nerve was stimulated to produce maximal force via the electrodes by delivering 0.2 square-wave pulses for 150 ms (Aurora Scientific Inc 701C: Electrical Stimulator). Peak isometric force was measured by stimulating at 200 Hz and manipulating voltage every minute until a plateau was attained (two contractions within 10 mN). The muscle length where maximal isometric twitch force (Pt) was produced was defined as the optimal muscle length (Lo). After defining Lo, muscle stimulation was performed at increasing frequencies with 45 s rest to avoid fatigue (1, 10, 20, 30, 40, 50, 60, 80, 100, 150, and 200 Hz). Peak force was used to define the absolute isometric tetanic force (P_o_). The specific force of the TA muscle was calculated using the cross-sectional area of the muscle, determined by measuring the muscle length with digital callipers from myotendinous junction to myotendinous junction, applying a fibre/length ratio of 0.6123, and considering the density of skeletal muscle (1.06 g·mL^−1^). One minute after the force frequency, the TA was subjected to a series of 40 eccentric contractions. Each eccentric contraction involved stimulating the TA at 150 Hz for 20 ms to achieve maximal force production. While being stimulated at 150 Hz, the muscle was lengthened 20% of its Lo over 100 ms, then passively returned to its Lo before the next contraction 10 s later. The decrease in force from the initial contraction was used to determine the sensitivity of muscle to eccentric contractions. Immediately after the final eccentric contraction, and at 1, 2, 5, 10, and 15 min, the TA was stimulated at 150 Hz to measure isometric tetanic force and recovery.

#### Bone morphology

Femora were thawed at room temperature and scanned by micro-computed tomography (SkyScan 1276, Bruker). First, bone samples were wrapped in cotton gauze soaked in phosphate buffered saline (PBS) to ensure stability within screw-cap tubes during scanning. The scanning was performed with a voxel resolution of 9 μm, using a 0.25 mm aluminum filter, 57 kV, 200 μA, and a 540 ms exposure time, with a 0.4° rotation and frame averaging of 2. Calibration rods were scanned simultaneously under the same settings. Image reconstruction was conducted using SkyScan NRecon software (version 1.7.4.6). Femoral length (mm) was measured using CT Analyzer (CTAn; version 1.18.8.0) and was used to define a metaphyseal and diaphyseal ROI to measure trabecular and cortical structures, respectively, in anatomically comparable regions, as described previously.^70^ The metaphyseal (trabecular) ROI started at 10% of femoral length proximal to the distal growth plate and extended proximally for 15% of femoral length. The cortical ROI began at 30% of femoral length proximal to the distal growth plate and extended proximally for 15% of femoral length. A lower threshold of 0.3034 mg/cm^3^ CaHA was used to define bone. Within the metaphyseal ROI, the following parameters were measured: trabecular bone volume (% total bone volume), trabecular thickness (μm), trabecular number (1/mm), and trabecular separation (μm). Within the diaphyseal ROI, a threshold of 0.6335 mg/cm^3^ CaHA was used to measure: cortical thickness (μm); marrow area (mm^2^), cortical area (μm^2^); endocortical perimeter (mm), and mean polar moment of inertia (mm^4^). Three-dimensional reconstructions were generated using ParaReview and CTVox, encompassing either the entire dataset of the femur or the first 50 slices (0.5 mm) of the analyzed ROI, which consisted of cross-sectional images. Any samples found to be broken or cracked within the ROIs for assessing cortical and trabecular bone were excluded (total of 6 samples).

#### Bone strength testing

After micro-CT imaging, femora from 24 week old mice were frozen at -20°C until mechanical testing. Femora from 24 week old mice were used to capture the cumulative effects of muscle pathology on bone health more effectively (*92*). Mechanical testing was performed using the three-point bending method with a Bose Biodynamic 5500 Test Instrument (Bose, Delaware), and data were collected with WinTest 7 software, as previously described.^80^ The bones were positioned horizontally, anterior surface upwards and centrally placed onto two supporting pins with a span width of 8 mm and the force applied vertically to the midshaft. The bones were loaded cranio-caudally at a speed of 0.5 mm/s to generate force-displacement curves and measure the bone properties. Force-displacement curves were normalized using cranio-caudal and medial-lateral diameter and cortical thickness of the midshaft, determined from micro-CT imaging, to calculate stress-strain curves and material-specific mechanical properties.^81^ Ultimate force was defined as maximum force attained, post-yield displacement as displacement from yield point to fracture point elastic modulus as slope of the stress-strain curve at the linear region of the curve, and stiffness was calculated as the slope of the linear (elastic) part of the load-displacement curve.^82^ A 0.2% strain offset was utilized on the stress-strain curve to ensure consistent identification of the yield point.^83^

### Quantification and statistical analysis

All data were assessed for normality using the Shapiro-Wilk test before conducting any analyses. For data following a normal distribution, either a one-, two- or three-way ANOVA were performed (stress paradigm, age, and genotype). Descriptive analysis was conducted with continuous outcome data reported as mean ± SD with statistical significance set at p < 0.05 for all tests. All data were analyzed and plotted using GraphPad Prism version 9 (GraphPad Software, USA) or Stata/SE 17 (StataCorp, TX, USA).

To analyze potential non-linear group trajectories in body mass, hemodynamic measurements, and Barnes maze parameters, generalized estimating equations, with exchangeable working correlation and robust standard errors, were used to fit cubic spline and polynomial models, which included all lower-order polynomial time and trial, effects and their interactions with group, allowing trajectories to vary across groups. We examined plots of the model-fitted and raw group means to ensure this model accurately represented the data. A *post-hoc* joint test of group by trial interactions (i.e., interactions involving trial polynomials) or group by time effects was used to examine differences in trajectories or slopes between groups. Significant differences in group curves were further explored using marginal analyses to assess group differences at specific time points. All models were adjusted for the baseline value of the response variable, which was not included as part of the outcome in the model.

To assess non-linear group trajectories specific to TA muscle isometric force over frequency, eccentric force over contraction, and isometric tetanic force over time, generalized estimating equations, with exchangeable working correlation and robust standard errors, the cubic polynomial model was employed. This model incorporated lower-order polynomial terms for time and contraction effects, along with their interactions with groups, allowing trajectories to vary across experimental conditions. Model accuracy was verified through examination of model-fitted and raw group means. Post-hoc joint tests of group by contraction interactions (i.e., involving contraction polynomials) or group by time effects were utilized to detect differences in trajectories or slopes between groups. Significant variations in group curves were further investigated using marginal analyses to evaluate group differences at specific time points. All models were adjusted for the baseline value of the response variable, which was not included as part of the outcome in the models.
