# Supplementary material for: Stress-induced fetal programming contributes to the manifestation of Duchenne muscular dystrophy in mdx mice
Source: iScience. 2025 Mar 1;28(4):112123. doi: 10.1016/j.isci.2025.112123 (PMC12131249; doi:10.1016/j.isci.2025.112123)
Supplement: Document S1. Figures S1–S6 and Tables S1–S16 [file mmc1.pdf]

iScience, Volume *28*

Supplemental information

Stress-induced fetal programming contributes to the manifestation of Duchenne muscular dystrophy in *mdx* mice

Saba Gharibi, Gretel S. Major, Ali Shad, Bridgette D. Semple, Narelle E. McGregor, Martha Blank, Gavin Abbott, Natalie A. Sims, Christopher S. Shaw, Aaron P. Russell, and Angus Lindsay

Supplemental material

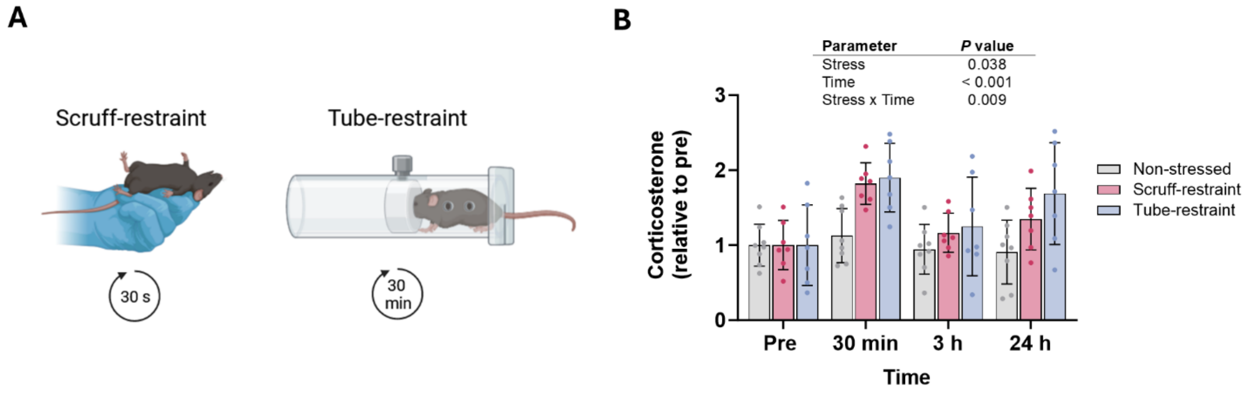

Fig. S1. The response of female C57BL/10-*mdx* heterozygous mice to stress paradigms. (A) Schematic representation of scruff-restraint and tube-restraint stress paradigms. **(B)** Relative serum corticosterone response of mice to scruff-restraint and tube-restraint stress paradigms. Data were analyzed using a repeated measures two-way ANOVA. N = 7 – 8/group. Data are mean ± SD.

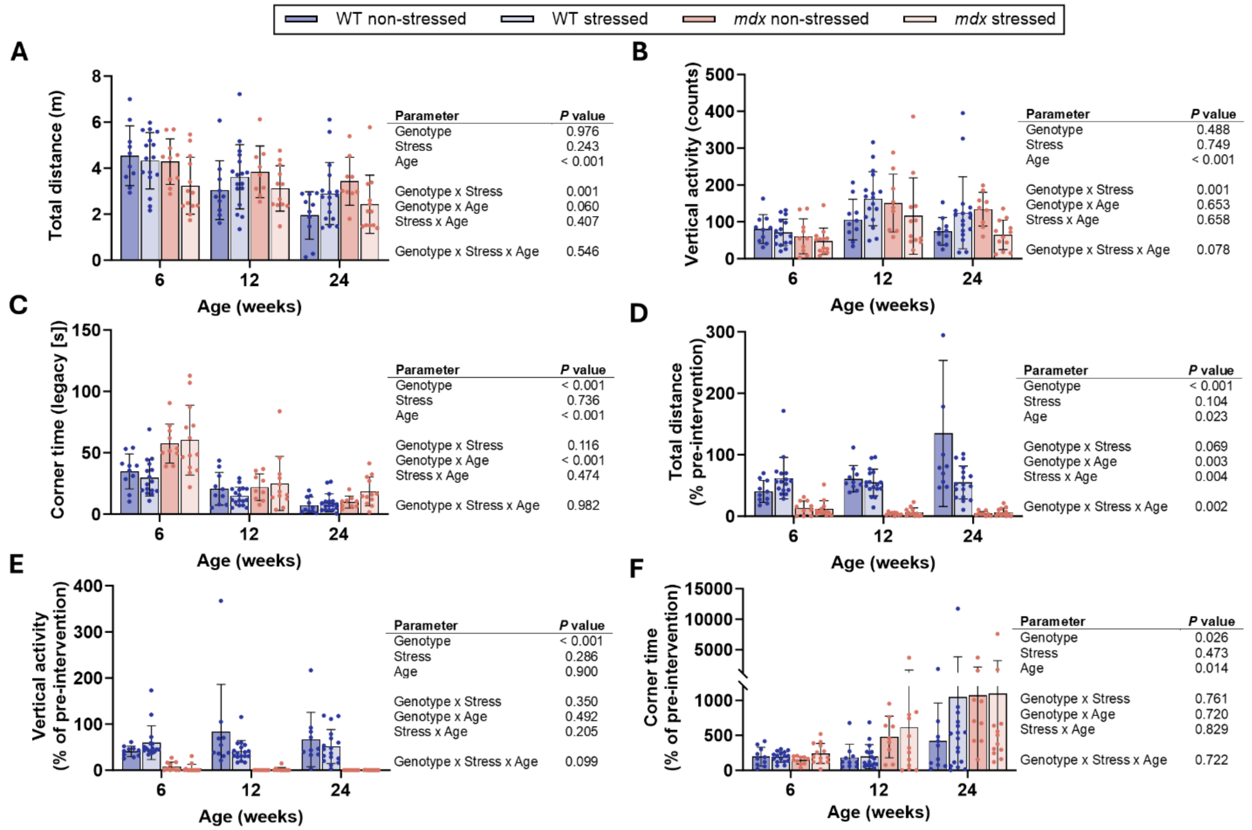

Fig. S2. The effect of gestational stress on open field physical activity and the acute stress response of offspring throughout development. Behavioral profiling of male wildtype (WT) and *mdx* mice born to female *mdx*-het mice that during the last week of gestation were either not stressed or stressed. (**A-C**) Exploratory open field physical activity and (**D-F**) open field physical activity after an acute 30 sec scruff-restraint stressor (data are presented as the ratio of post-stressor physical activity to pre-stressor physical activity (% of intervention)). (**A**), (**D**) Total distance ambulated, (**B**), (**E**) vertical activity and (**C**), (**F**) time spent in the corner (left-front, right-front, left-rear, right-rear). All data were analyzed using three-way ANOVA. N = 10 – 17/genotype/group. All data are mean ± SD.

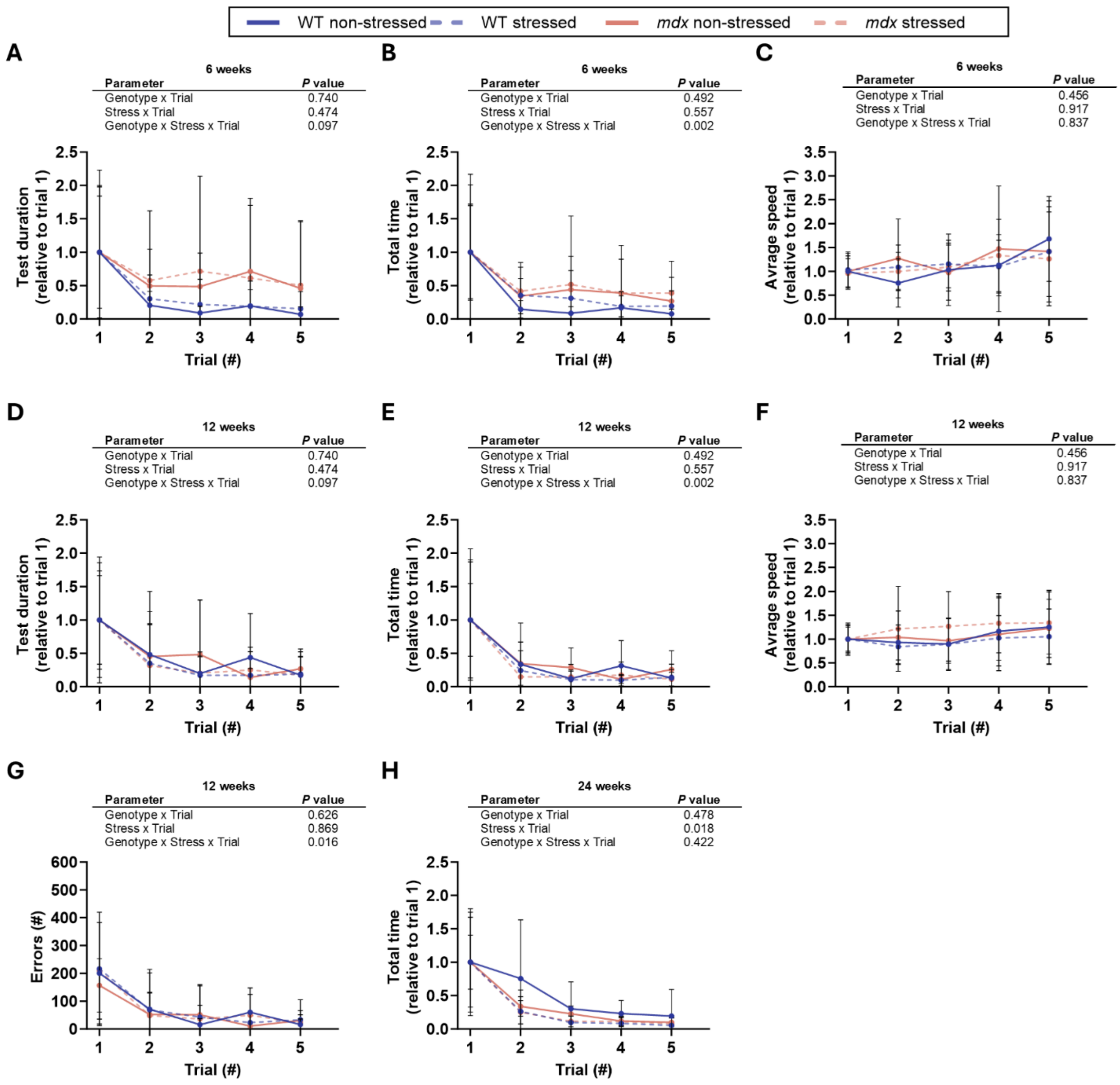

Fig. S3. The effect of gestational stress on the memory of offspring throughout development in a Barnes maze. Analysis of male wildtype (WT) and *mdx* mice born to female *mdx*-het mice that during the last week of gestation were either not stressed or stressed. (**A**), (**D**) Duration of the test, (**B**), (**E**) and (**H**) the length of time the animal was mobile during the test. (**C**), (**F**) the average speed of mice during the test and (**G**) the total number of errors encountered in locating the escape hole. Data were analyzed using cubic polynomial model. N = 10 – 17/genotype/group. All data are mean ± SD.

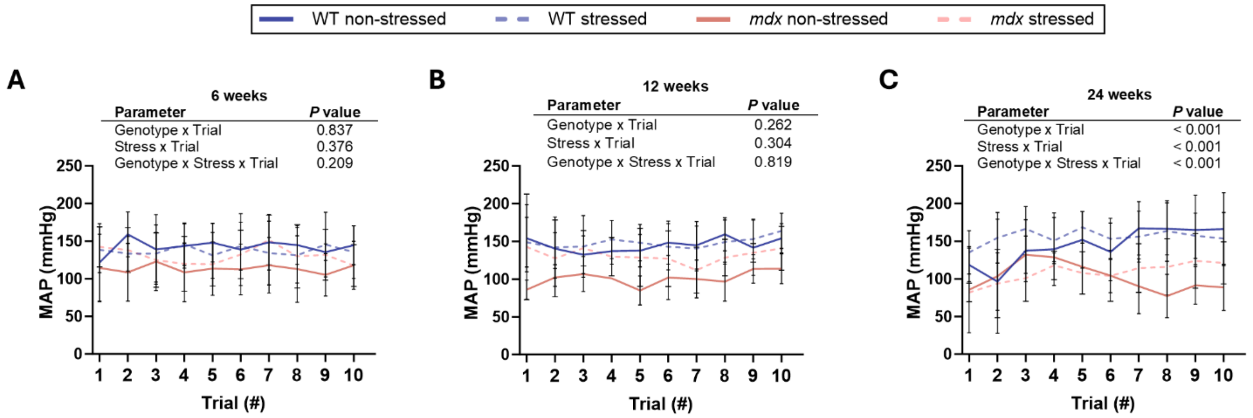

Fig. S4. The effect of gestational stress on mean arterial pressure (MAP) profiles of offspring throughout development. Repeated arterial pressure measurements throughout a 5-min tube-restraint stressor of male wildtype (WT) and *mdx* mice born to female *mdx*-het mice that during the last week of gestation were either not stressed or stressed. Profiles at (**A**) 6, (**B**) 12 and (**C**) 24 weeks of age. Data were analyzed using cubic polynomial model. N = 10 – 17/genotype/group. All data are mean ± SD.

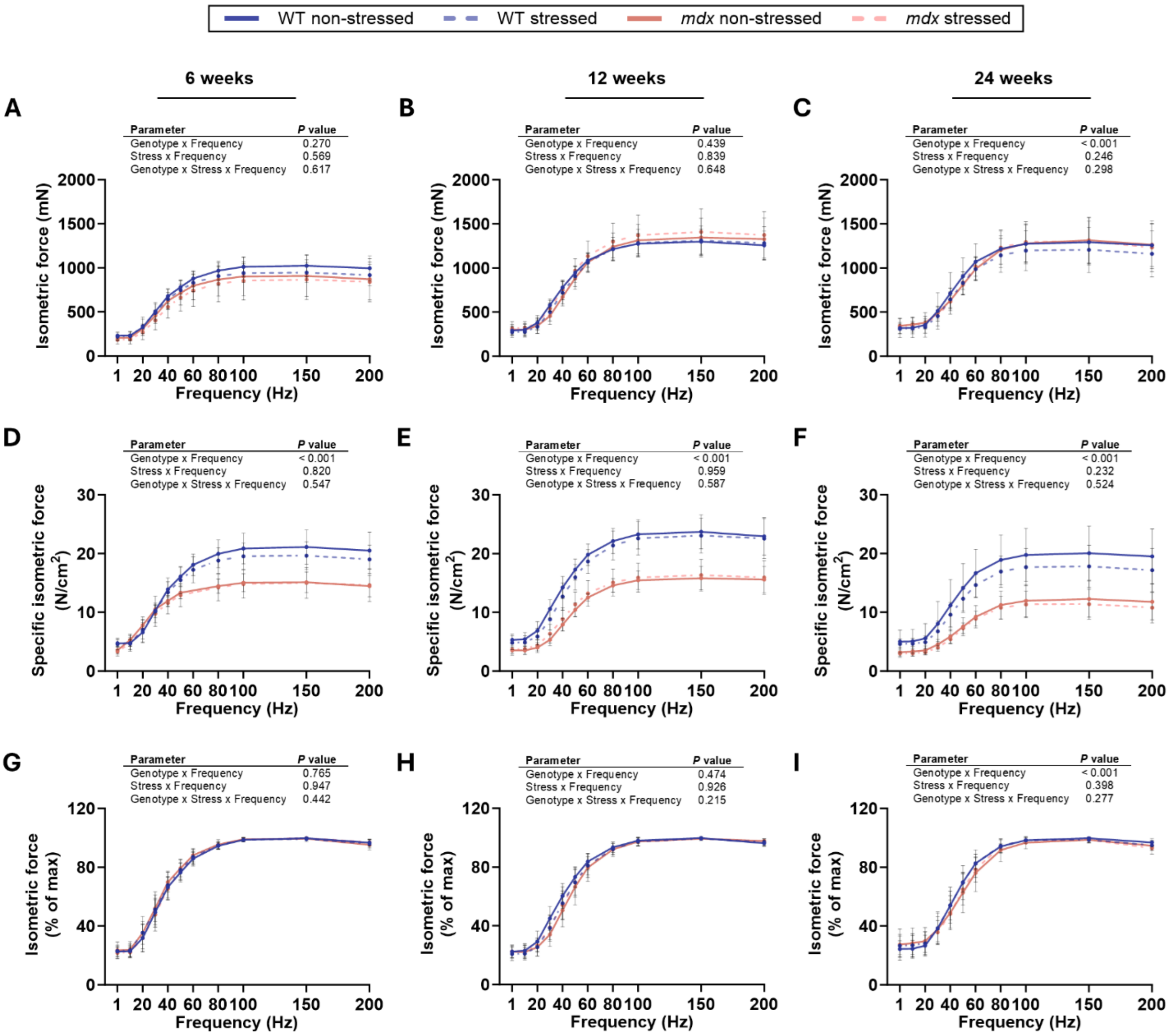

Fig. S5. The effect of gestational stress on muscle force production in offspring throughout development. Analysis of force production in the tibialis anterior muscles from male wildtype (WT) and *mdx* mice born to female *mdx*-het mice that during the last week of gestation were either not stressed or stressed. (**A**), (**B**), (**C**) Force frequency curves, (**D**), (**E**), (**F**) specific force frequency curves, and (**G**), (**H**), (**I**) force frequency as a percentage of maximum force at 6, 12 and 24 weeks of age, respectively. Data were analyzed using a cubic polynomial model. N = 9 – 16/genotype/group. All data are mean ± SD.

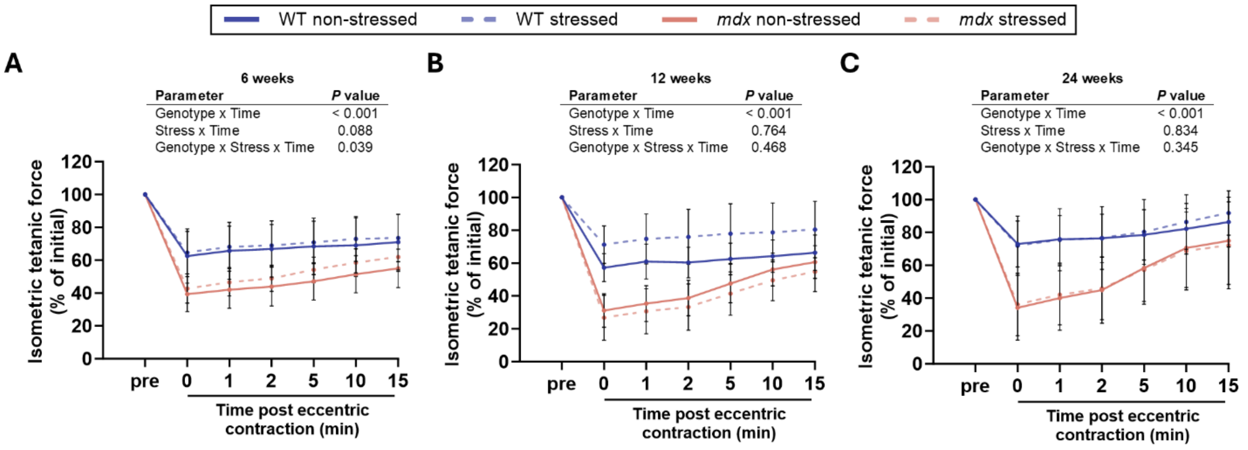

Fig. S6. The effect of gestational stress on force recovery after eccentric contraction in offspring throughout development. Percentage force recovery profiles of the tibialis anterior muscles up to 15 minutes post-eccentric contraction in offspring at (**A**) 6, (**B**) 12 and (**C**) 24 weeks of age. Male wildtype (WT) and *mdx* mice were born to female *mdx*-het mice that during the last week of gestation were either not stressed or stressed. Data were analyzed using three-way ANOVA. N = 9 – 17/genotype/group. Data are mean ± SD.

Table S1. The mass of organs from male wildtype and *mdx* mice born to non-stressed or stressed *mdx*-heterozygous mothers at 6, 12 and 24 weeks of age. Values are mean ± SD. Three different stress paradigms were employed including a non-stressed condition (NS), a 30-second scruff restraint (SR), and a 30-minute tube restraint (TR).

Parameter	Age (weeks)	Wildtype			*mdx*			Genotype (*p* value)	Paradigm (*p* value)	Time (*p* value)	Genotype x Paradigm (*p* value)	Genotype x Time (*p* value)	Paradigm x Time (*p* value)	Genotype x Paradigm x Time (*p* value)
		NS	SR	TR	NS	SR	TR							
**Adrenal mass (mg)**	6	2.26 ± 0.29	2.17 ± 0.30	2.23 ± 0.11	2.56 ± 0.38	2.44 ± 0.27	2.34 ± 0.22	**< 0.001**	0.858	**0.075**	0.718	0.424	0.475	0.961
	12	2.19 ± 0.27	2.29 ± 0.34	2.32 ± 0.25	2.61 ± 0.20	2.67 ± 0.34	2.66 ± 0.35							
	24	2.29 ± 0.19	2.33 ± 0.27	2.26 ± 0.26	2.56 ± 0.26	2.67 ± 0.20	2.55 ± 0.32							
**Adrenal mass (% of body mass)**	6	0.010 ± 0.001	0.009 ± 0.001	0.010 ± 0.001	0.012 ± 0.001	0.012 ± 0.002	0.011 ± 0.002	**< 0.001**	0.703	**< 0.001**	0.447	0.158	0.403	0.628
	12	0.007 ± 0.001	0.008 ± 0.001	0.008 ± 0.001	0.009 ± 0.001	0.008 ± 0.000	0.008 ± 0.001							
	24	0.006 ± 0.001	0.005 ± 0.001	0.005 ± 0.001	0.007 ± 0.001	0.006 ± 0.001	0.007 ± 0.001							
**Heart mass (mg)**	6	121 ± 11.3	119 ± 11.8	111 ± 6.56	109 ± 14.6	107 ± 15.1	106 ± 7.89	0.700	0.272	**< 0.001**	0.683	**0.032**	0.486	0.986
	12	140 ± 14.1	145 ± 15.1	139 ± 12.6	137 ± 19.8	146 ± 33.1	143 ± 15.5							
	24	159 ± 11.8	166 ± 12.6	163 ± 9.97	164 ± 11.2	173 ± 15.0	169 ± 11.3							
**Heart mass (% of body mass)**	6	0.527 ± 0.029	0.519 ± 0.036	0.510 ± 0.020	0.517 ± 0.028	0.502 ± 0.026	0.512 ± 0.045	0.153	0.989	**< 0.001**	0.160	**< 0.001**	0.403	0.902
	12	0.453 ± 0.038	0.476 ± 0.048	0.457 ± 0.021	0.442 ± 0.027	0.450 ± 0.041	0.447 ± 0.025							
	24	0.382 ± 0.034	0.387 ± 0.020	0.373 ± 0.020	0.429 ± 0.029	0.413 ± 0.013	0.445 ± 0.041							
**Brain mass (mg)**	6	438 ± 8.28	434 ± 9.39	416 ± 21.7	414 ± 21.9	418 ± 15.8	412 ± 17.3	**0.026**	**0.053**	**< 0.001**	0.441	**0.066**	0.162	0.456
	12	429 ± 18.2	439 ± 13.9	431 ±12.4	433 ± 24.2	436 ± 27.5	430 ± 16.1							
	24	449 ± 6.72	454 ± 11.6	452 ± 12.5	437 ± 13.6	457 ± 17.9	451 ± 13.2							
**Brain mass (% of body mass)**	6	1.916 ± 0.152	1.906 ± 0.169	1.918 ± 0.052	1.992 ± 0.236	1.979 ± 0.204	1.985 ± 0.127	**0.082**	0.900	**< 0.001**	0.673	**0.039**	0.923	0.684
	12	1.388 ± 0.099	1.444 ± 0.077	1.421 ± 0.074	1.406 ± 0.962	1.374 ± 0.155	1.351 ± 0.082							
	24	1.081 ± 0.068	1.063 ± 0.082	1.038 ± 0.069	1.144 ± 0.071	1.094 ± 0.075	1.190 ± 0.145							

Table S2. Open-field physical activity (10 min assay) of male wildtype and *mdx* offspring born to non-stressed or stressed *mdx*-heterozygous mothers at 6, 12 and 24 weeks of age. Values are mean ± SD. Three different stress paradigms were employed including a non-stressed condition (NS), a 30-second scruff restraint (SR), and a 30-minute tube restraint (TR).

**Parameter**	**Age (weeks)**	**Wildtype**			***mdx***			**Genotype** (*p* value)	**Paradigm** (*p* value)	**Time** (*p* value)	**Genotype x Paradigm** (*p* value)	**Genotype x Time** (*p* value)	**Paradigm x Time** (*p* value)	**Genotype x Paradigm x Time** (*p* value)
		NS	SR	TR	NS	SR	TR							
**Total distance (m)**	6	4.54 ± 1.30	4.20 ± 1.35	4.54 ± 1.05	4.28 ± 0.99	2.85 ± 0.87	368 ± 151	**0.314**	0.435	**< 0.001**	0.006	0.109	0.222	0.743
	12	3.04 ± 1.27	3.99 ± 1.43	3.10 ± 1.25	3.85 ± 1.12	3.21 ± 0.93	302 ± 112							
	24	1.95 ± 1.03	2.99 ± 1.12	2.74 ± 1.81	3.43 ± 1.04	2.82 ± 1.61	204 ± 75.0							
**Movement time (s)**	6	334 ± 55.5	304 ± 52.8	307 ± 38.7	273 ± 48.8	172 ± 47.8	240 ± 78.5	**< 0.001**	**0.037**	**0.007**	**< 0.001**	**0.025**	**0.059**	0.204
	12	305 ± 45.5	374 ± 64.6	296 ± 71.9	316 ± 59.0	269 ± 88.9	315 ± 70.9							
	24	284 ± 48.1	323 ± 65.8	291 ± 103	339 ± 62.2	258.0 ± 30.7	224 ± 72.3							
**Vertical activity (counts)**	6	80.5 ± 39.2	77.5 ± 36.3	59.8 ± 36.5	60.5 ± 47.4	33.7 ± 19.8	63.0 ± 45.2	0.109	0.409	**< 0.001**	0.007	0.958	0.638	0.157
	12	106 ± 55.3	186 ± 63.6	123 ± 78.4	151 ± 78.5	127 ± 134	105 ± 72.8							
	24	74.2 ± 37.6	125 ± 71.2	124 ± 83.0	135 ± 45.9	86.2 ± 30.6	43.2 ± 38.1							
**Ambulatory episode average velocity (m/s)**	6	0.062 ± 0.008	0.074 ± 0.026	0.061 ± 0.010	0.056 ± 0.009	0.056 ± 0.010	0.052 ± 0.004	**< 0.001**	**0.097**	**0.038**	**0.015**	0.830	0.766	0.646
	12	0.062 ± 0.010	0.068 ± 0.008	0.063 ± 0.011	0.055 ± 0.006	0.053 ± 0.007	0.051 ± 0.005							
	24	0.055 ± 0.009	0.062 ± 0.006	0.051 ± 0.004	0.056 ± 0.008	0.049 ± 0.006	0.046 ± 0.003							
**Ambulatory episode peak average velocity (m /s)**	6	0.185 ± 0.031	0.211 ± 0.083	0.186 ± 0.044	0.179 ± 0.065	0.164 ± 0.035	0.144 ± 0.023	**< 0.001**	0.293	0.299	0.091	0.616	0.859	0.966
	12	0.174 ± 0.031	0.208 ± 0.052	0.198 ± 0.050	0.155 ± 0.022	0.154 ± 0.041	0.151 ± 0.031							
	24	0.162 ± 0.037	0.196 ± 0.034	0.164 ± 0.033	0.157 ± 0.034	0.149 ± 0.039	0.156 ± 0.078							
**Corner time (legacy; s)**	6	34.9 ± 14.2	30.3 ± 17.1	8.81 ± 9.14	57.6 ± 15.9	29.0 ± 28.6	67.7 ± 22.1	**< 0.001**	0.903	**< 0.001**	0.271	**< 0.001**	0.428	0.592
	12	20.8 ± 13.3	14.8 ± 8.10	67.7 ± 22.2	22.0 ± 10.8	22.7 ± 13.1	21.1 ± 14.5							
	24	7.48 ± 6.58	9.56 ± 6.72	21.1 ± 14.5	9.91 ± 4.83	28.7 ± 11.8	14.3 ± 9.66							

	6	434 ± 78.4	374 ± 85.4	441 ± 62.2	515 ± 53.7	423 ± 166	534 ± 42.3							
**Margin time (legacy; s)**	12	337 ± 61.5	285 ± 47.2	324 ± 50.3	357 ± 80.4	418 ± 86.6	344 ± 115	**< 0.001**	0.872	**< 0.001**	0.349	0.521	**0.015**	0.191
	24	229 ± 121	255 ± 94.8	179 ± 117	273 ± 51.1	349 ± 69.1	340 ± 107							
	6	166 ± 78.4	196 ± 78.6	159 ± 62.2	85.2 ± 53.7	48.3 ± 43.9	66.5 ± 42.3							
**Centre time (legacy; s)**	12	263 ± 61.5	315 ± 47.2	276 ± 50.3	243 ± 80.5	182 ± 86.6	256 ± 115	**< 0.001**	0.412	**< 0.001**	**0.049**	**0.283**	0.606	0.306
	24	371 ± 121	345 ± 94.8	421 ± 117	327 ± 51.1	250 ± 69.1	260 ± 107							

Table S3. Open field physical activity of male wildtype and *mdx* offspring born to non-stressed or stressed *mdx*-heterozygous mothers in response to a 30-second scruff restraint stressor at 6, 12 and 24 weeks of age. Values are mean ± SD. Three different stress paradigms were employed including a non-stressed condition (NS), a 30-second scruff restraint (SR), and a 30-minute tube restraint (TR).

Parameter	Age (weeks)	Wildtype			*mdx*			Genotype (*p* value)	Paradigm (*p* value)	Time (*p* value)	Genotype x Paradigm (*p* value)	Genotype x Time (*p* value)	Paradigm x Time (*p* value)	Genotype x Paradigm x Time (*p* value)
		NS	SR	TR	NS	SR	TR							
**Total distance (% of intervention)**	6	40.4 ± 18.0	68.3 ± 40.5	51.7 ± 15.9	13.4 ± 11.5	14.7 ± 18.2	8.30 ± 5.20	**< 0.001**	0.264	0.239	0.188	**0.078**	**0.026**	**0.016**
	12	61.0 ± 21.4	49.5 ± 20.2	62.7 ± 22.9	3.93 ± 2.45	8.65 ± 9.50	4.61 ± 3.30							
	24	135 ± 119	54.5 ± 22.8	55.5 ± 35.6	4.10 ± 3.63	8.28 ± 8.17	4.85 ± 5.36							
**Movement time (% of intervention)**	6	58.1 ± 10.90	78.1 ± 29.0	70.4 ± 3.8	21.8 ± 14.3	30.9 ± 30.1	12.7 ± 5.53	**< 0.001**	0.180	0.195	0.585	**0.013**	0.287	0.212
	12	78.4 ± 11.24	69.1 ± 10.5	79.0 ± 16.9	7.93 ± 5.09	14.6 ± 7.39	5.41 ± 1.69							
	24	76.4 ± 22.4	73.7 ± 28.6	63.4 ± 24.0	4.59 ± 2.27	9.38 ± 11.5	5.56 ± 3.82							
**Vertical activity (% of intervention)**	6	40.9 ± 11.3	44.6 ± 7.9	68.8 ± 44.1	7.84 ± 9.8	6.53 ± 12.1	0.45 ± 1.11	**< 0.001**	0.586	0.989	0.624	0.732	0.325	0.252
	12	83.1 ± 103	46.6 ± 33.0	37.8 ± 14.1	0.05 ± 0.13	3.21 ± 5.79	0.0 ± 0.0							
	24	66.4 ± 59.0	65.4 ± 45.6	43.8 ± 30.7	0.0 ± 0.0	0.12 ± 0.30	0.0 ± 0.0							
**Ambulatory episode average velocity (% of intervention)**	6	79.2 ± 11.3	74.9 ± 21.3	81.8 ± 11.9	57.6 ± 31.6	63.2 ± 10.1	63.1 ± 17.6	**< 0.001**	0.281	0.446	0.586	0.100	0.352	0.693
	12	85.7 ± 17.7	79.0 ± 15.0	82.2 ± 15.4	69.2 ± 18.2	52.0 ± 31.9	79.3 ± 38.7							
	24	92.1 ± 11.2	86.7 ± 9.74	79.2 ± 11.5	58.9 ± 15.4	50.4 ± 29.1	51.7 ± 31.4							
**Ambulatory episode peak average velocity (% of intervention)**	6	92.3 ± 50.8	79.2 ± 22.2	84.9 ± 35.3	43.5 ± 32.6	44.3 ± 18.5	42.7 ± 22.2	**< 0.001**	**0.089**	0.686	0.667	0.312	0.894	0.927
	12	96.9 ± 19.3	86.2 ± 24.3	74.8 ± 14.9	48.3 ± 19.5	43.0 ± 38.5	45.1 ± 26.4							
	24	100 ± 25.1	80.7 ± 21.1	86.5 ± 21.7	42.2 ± 18.1	28.9 ± 22.6	27.5 ± 22.2							
**Corner time (legacy; % of intervention)**	6	196 ± 134	196.4 ± 69.6	184 ± 90.1	146 ± 55.1	295 ± 176	178 ± 57.2	0.457	0.149	**0.001**	0.639	0.709	**0.008**	0.807
	12	185 ± 187	215 ± 205	171 ± 120	478 ± 298	965 ± 1367	263 ± 310							
	24	421 ± 542	334 ± 252	2356 ± 4604	1069 ± 1067	316 ± 179	1877 ± 2837							

**Margin time (legacy; % of intervention)**	6	111 ± 21.9	122 ± 36.5	99.4 ± 13.9	113 ± 14.0	165 ± 71.9	112 ± 8.22	**0.136**	0.151	**< 0.001**	0.128	0.246	**0.021**	**0.006**
	12	116 ± 18.9	131 ± 39.6	124 ± 29.05	159 ± 70.1	149 ± 39.02	191 ± 61.7							
	24	142 ± 63.4	150 ± 54.8	319 ± 278	226 ± 50.3	174 ± 31.6	189 ± 45.0							
**Center time (legacy; % of intervention)**	6	83.1 ± 49.7	106 ± 71.2	119 ± 47.8	28.4 ± 47.1	3.12 ± 7.03	3.86 ± 6.18	**< 0.001**	0.893	**0.027**	0.618	0.504	0.933	0.141
	12	81.2 ± 23.8	78.8 ± 31.3	76.4 ± 29.9	1.81 ± 3.01	3.18 ± 4.45	0.78 ± 0.66							
	24	89.8 ± 28.7	72.6 ± 29.8	72.8 ± 55.8	2.29 ± 6.51	4.05 ± 4.00	0.78 ± 1.92							

Table S4. Physiological parameters of cardiovascular function during 5 min tube-restraint from wildtype and *mdx* offspring born to non-stressed or stressed *mdx*-heterozygous mothers at 6, 12 and 24 weeks of age. Values are mean ± SD. MAP = Mean arterial blood pressure. Shock index is calculated by maximum heart rate/low systolic blood pressure. Three different stress paradigms were employed including a non-stressed condition (NS), a 30-second scruff restraint (SR), and a 30-minute tube restraint (TR).

**Parameter**	**Age (weeks)**	**Wildtype**			***mdx***			**Genotype** (*p* value)	**Paradigm** (*p* value)	**Time** (*p* value)	**Genotype x Paradigm** (*p* value)	**Genotype x Time** (*p* value)	**Paradigm x Time** (*p* value)	**Genotype x Paradigm x Time** (*p* value)
		NS	SR	TR	NS	SR	TR							
**MAP (mmHg)**	6	144 ± 23.7	135 ± 25.2	139 ± 23.9	113 ± 29.1	123 ± 29.8	131 ± 35.6	**< 0.001**	0.123	0.636	0.324	**0.013**	0.886	0.753
	12	146 ± 23.2	151 ± 26.0	146 ± 18.4	105 ± 16.9	126 ± 26.9	133 ± 26.1							
	24	146 ± 26.0	159 ± 23.3	157 ± 17.9	100 ± 27.9	108 ± 25.8	108 ± 31.7							
**Heart rate (bpm)**	6	622 ± 40.9	645 ± 74.0	623 ± 45.2	650 ± 38.3	638 ± 34.0	592 ± 34.6	**0.597**	**0.007**	**< 0.001**	**0.065**	0.777	0.825	0.272
	12	662 ± 75.1	656 ± 44.0	647 ± 20.1	702 ± 62.6	673 ± 32.2	625 ± 50.3							
	24	667 ± 28.5	692 ± 23.1	640 ± 52.3	692 ± 81.6	651 ± 52.6	672 ± 27.5							
**Shock index**	6	4.81 ± 1.19	5.30 ± 1.39	4.71 ± 0.81	6.41 ± 1.81	5.51 ± 1.61	5.04 ± 1.80	**< 0.001**	**0.006**	0.832	0.372	0.565	0.688	0.743
	12	5.21 ± 1.71	4.75 ± 1.35	4.78 ± 0.49	6.64 ± 1.20	5.47 ± 1.22	5.30 ± 1.74							
	24	5.56 ± 1.43	4.47 ± 0.50	4.38 ± 0.68	6.76 ± 2.09	5.51 ± 0.86	6.13 ± 1.91							

Table S5. The mass and length of muscle and bone from male wildtype and *mdx* mice born to non-stressed or stressed *mdx*-heterozygous mothers at 6, 12 and 24 weeks of age. Values are mean ± SD. Three different stress paradigms were employed including a non-stressed condition (NS), a 30-second scruff restraint (SR), and a 30-minute tube restraint (TR).

Parameter	Age (weeks)	Wildtype			*mdx*			Genotype (*p* value)	Paradigm (*p* value)	Time (*p* value)	Genotype x Paradigm (*p* value)	Genotype x Time (*p* value)	Paradigm x Time (*p* value)	Genotype x Paradigm x Time (*p* value)
		NS	SR	TR	NS	SR	TR							
**Tibialis anterior mass (mg)**	6	37.3 ± 6.40	36.5 ± 4.13	34.4 ± 5.39	42.2 ± 9.22	43.3 ± 1.67	38.3 ± 8.24	**< 0.001**	0.022	**< 0.001**	0.315	**< 0.001**	0.367	0.933
	12	42.9 ± 3.42	44.5 ± 3.09	43.1 ± 2.26	67.5 ± 8.65	70.5 ± 12.6	66.8 ± 6.55							
	24	49.4 ± 2.52	53.3 ± 4.98	51.9 ± 2.66	87.1 ± 5.01	96.3 ± 8.32	86.6 ± 11.6							
**Tibialis anterior mass (% of body mass)**	6	0.162 ± 0.022	0.159 ± 0.008	0.158 ± 0.020	0.198 ± 0.017	0.200 ± 0.027	0.182 ± 0.028	**< 0.001**	0.173	0.477	0.480	**< 0.001**	0.843	0.835
	12	0.139 ± 0.016	0.146 ± 0.006	0.142 ± 0.008	0.218 ± 0.018	0.219 ± 0.014	0.208 ± 0.015							
	24	0.119 ± 0.010	0.124 ± 0.008	0.120 ± 0.008	0.228 ± 0.018	0.230 ± 0.011	0.227 ± 0.031							
**Gastrocnemius mass (mg)**	6	127 ± 14.2	131 ± 14.1	120 ± 10.4	123 ± 22.5	122 ± 24.9	116 ± 11.5	**< 0.001**	0.390	**< 0.001**	0.818	**< 0.001**	0.552	0.993
	12	153 ± 14.1	164 ± 7.48	163 ± 10.3	185 ± 34.9	191 ± 42.5	192 ± 16.9							
	24	181 ± 8.82	187 ± 12.0	185 ± 7.47	202 ± 15.0	207 ± 21.8	212 ± 15.3							
**Gastrocnemius mass (% of body mass)**	6	0.551 ± 0.032	0.568 ± 0.024	0.551 ± 0.026	0.579 ± 0.030	0.564 ± 0.048	0.558 ± 0.034	**< 0.001**	0.562	**< 0.001**	**0.013**	**< 0.001**	0.108	0.220
	12	0.495 ± 0.038	0.538 ± 0.018	0.536 ± 0.012	0.594 ± 0.053	0.588 ± 0.059	0.600 ± 0.030							
	24	0.435 ± 0.024	0.437 ± 0.018	0.424 ± 0.018	0.528 ± 0.036	0.493 ± 0.042	0.557 ± 0.058							
**Soleus mass (mg)**	6	6.31 ± 0.77	6.37 ± 0.99	6.13 ± 0.46	7.30 ± 1.54	7.34 ± 1.19	6.83 ± 1.41	**< 0.001**	0.910	**< 0.001**	0.944	**< 0.001**	0.279	0.999
	12	8.40 ± 1.19	7.97 ± 0.98	8.07 ± 1.33	11.9 ± 1.46	11.5 ± 3.11	11.6 ± 1.15							
	24	10.7 ± 1.17	11.4 ± 0.80	11.7 ± 1.47	15.0 ± 1.21	15.7 ± 1.23	15.8 ± 1.20							
**Soleus mass (% of body mass)**	6	0.027 ± 0.003	0.028 ± 0.004	0.028 ± 0.002	0.034 ± 0.005	0.034 ± 0.003	0.032 ± 0.004	**< 0.001**	0.520	**0.022**	0.505	**< 0.001**	0.435	0.683
	12	0.027 ± 0.003	0.026 ± 0.002	0.026 ± 0.003	0.038 ± 0.004	0.035 ± 0.005	0.036 ± 0.002							
	24	0.026 ± 0.003	0.034 ± 0.002	0.027 ± 0.003	0.039 ± 0.005	0.038 ± 0.005	0.041 ± 0.005							
**Tibia mass (mg)**	6	44.2 ± 4.52	46.8 ± 5.10	45.7 ± 7.63	40.9 ± 7.94	40.0 ± 6.02	39.3 ± 4.02	**0.014**	**0.016**	**< 0.001**	0.841	0.148	0.187	0.841
	12	54.1 ± 4.93	55.7 ± 6.49	51.2 ± 5.51	51.3 ± 7.45	53.9 ± 11.7	52.0 ± 7.67							
	24	66.0 ± 3.81	73.2 ± 6.55	68.2 ± 2.75	64.1 ± 3.89	71.8 ± 6.93	68.9 ± 4.51							

**Tibia mass (% of body mass)**	6	0.190 ± 0.012	0.204 ± 0.018	0.210 ± 0.027	0.194 ± 0.028	0.188 ± 0.024	0.189 ± 0.023	0.232	0.363	**< 0.001**	0.232	**0.005**	0.588	0.311
	12	0.176 ± 0.026	0.182 ± 0.016	0.168 ± 0.010	0.165 ± 0.014	0.166 ± 0.014	0.162 ± 0.015							
	24	0.160 ± 0.013	0.171 ± 0.012	0.158 ± 0.011	0.168 ± 0.013	0.171 ± 0.009	0.181 ± 0.016							
**Femur mass (mg)**	6	55.3 ± 6.00	56.1 ± 8.37	52.9 ± 4.31	45.9 ± 9.22	47.3 ± 5.45	42.0 ± 5.30	**< 0.001**	0.305	**< 0.001**	0.866	**0.010**	0.405	0.955
	12	68.5 ± 4.17	69.7 ± 7.52	70.7 ± 5.68	64.9 ± 10.1	67.5 ± 12.6	68.7 ± 6.58							
	24	90 ± 7.43	94.4 ± 6.69	95.3 ± 4.99	91.0 ± 3.40	93.8 ± 9.80	92.0 ± 5.94							
**Femur mass (% of body mass)**	6	0.240 ± 0.016	0.244 ± 0.020	0.244 ± 0.014	0.216 ± 0.019	0.223 ± 0.024	0.202 ± 0.013	**< 0.001**	0.843	**0.031**	0.432	**< 0.001**	0.173	0.266
	12	0.221 ± 0.018	0.228 ± 0.018	0.232 ± 0.015	0.209 ± 0.014	0.209 ± 0.015	0.215 ± 0.005							
	24	0.216 ± 0.017	0.220 ± 0.011	0.219 ± 0.022	0.238 ± 0.011	0.223 ± 0.012	0.242 ± 0.022							
**Tibia length (mm)**	6	15.8 ± 0.36	16.1 ± 0.42	16.0 ± 0.72	15.8 ± 0.97	15.4 ± 0.47	15.7 ± 0.57	0.906	0.369	**< 0.001**	0.243	0.151	0.420	0.281
	12	17.4 ± 0.47	17.4 ± 0.48	16.9 ± 0.38	17.2 ± 0.67	17.3 ± 0.59	17.5 ± 0.55							
	24	18.5 ± 0.46	18.8 ± 0.56	18.6 ± 0.76	18.5 ± 0.75	19.1 ± 0.69	19.0 ± 0.79							
**Femur length (mm)**	6	13.5 ± 0.60	13.2 ± 1.06	13.8 ± 0.95	12.8 ± 1.03	13.3 ± 0.80	12.4 ± 0.78	0.650	0.328	**< 0.001**	0.679	**< 0.001**	0.793	**0.031**
	12	15.1 ± 0.51	15.3 ± 0.60	15.1 ± 0.82	14.8 ± 0.75	15.2 ± 0.43	15.6 ± 0.14							
	24	16.1 ± 0.51	16.6 ± 0.60	16.4 ± 0.11	16.9 ± 0.58	16.8 ± 0.26	16.9 ± 0.39							
**Tibia mass (Of tibia length; mg/mm)**	6	2.807 ± 0.247	2.920 ± 0.344	2.851 ± 0.366	2.581 ± 0.427	2.587 ± 0.325	2.494 ± 0.216	**0.003**	**0.036**	**< 0.001**	0.951	0.189	0.482	0.974
	12	3.110 ± 0.247	3.192 ± 0.318	3.029 ± 0.269	2.980 ± 0.356	3.105 ± 0.587	2.964 ± 0.379							
	24	3.549 ± 0.248	3.890 ± 0.343	3.688 ± 0.266	3.470 ± 0.117	3.756 ± 0.257	3.633 ± 0.281							
**Femur mass (Of femur length; mg/mm)**	6	4.092 ± 0.347	4.156 ± 0.462	3.835 ± 0.122	3.566 ± 0.528	3.557 ± 0.269	3.390 ± 0.230	**< 0.001**	0.676	**< 0.001**	0.848	0.127	0.452	0.916
	12	4.546 ± 0.278	4.556 ± 0.388	4.690 ± 0.212	4.376 ± 0.572	4.412 ± 0.723	4.356 ± 0.419							
	24	5.566 ± 0.521	5.685 ± 0.380	5.815 ± 0.295	5.393 ± 0.234	5.567 ± 0.511	5.455 ± 0.369							

Table S6. Physiological parameters of isolated tibialis anterior from male wildtype and *mdx* offspring born to non-stressed or stressed *mdx*-heterozygous mothers at 6, 12 and 24 weeks of age. Values are mean ± SD. CSA = Cross-sectional area. ECC = eccentric contractions. Three different stress paradigms were employed including a non-stressed condition (NS), a 30-second scruff restraint (SR), and a 30-minute tube restraint (TR).

**Parameter**	**Age (weeks)**	**Wildtype**			***mdx***			**Genotype** (*p* value)	**Paradigm** (*p* value)	**Time** (*p* value)	**Genotype x Paradigm** (*p* value)	**Genotype x Time** (*p* value)	**Paradigm x Time** (*p* value)	**Genotype x Paradigm x Time** (*p* value)
		NS	SR	TR	NS	SR	TR							
**CSA (cm^2)**	6	0.05 ± 0.01	0.05 ± 0.00	0.05 ± 0.00	0.06 ± 0.01	0.06 ± 0.01	0.05 ± 0.01	**< 0.001**	0.529	**< 0.001**	0.412	**< 0.001**	0.266	0.814
	12	0.06 ± 0.01	0.06 ± 0.00	0.06 ± 0.00	0.09 ± 0.01	0.09 ± 0.01	0.09 ± 0.01							
	24	0.07 ± 0.01	0.07 ± 0.01	0.07 ± 0.00	0.10 ± 0.01	0.12 ± 0.00	0.11 ± 0.01							
**Isometric twitch force (mN)**	6	233 ± 41.4	233 ± 46.2	212 ± 66.1	203 ± 38.5	202 ± 64.1	174 ± 36.4	**0.301**	0.289	0.803	0.803	**0.018**	0.759	0.891
	12	299 ± 62.5	275 ± 61.2	281 ± 63.9	299 ± 71.0	323 ± 70.8	324 ± 76.0							
	24	323 ± 113	331 ± 55.7	284 ± 61	359 ± 84.8	362 ± 53.4	330 ± 40.5							
**Specific isometric twitch force (N/cm^2)**	6	4.76 ± 0.59	4.79 ± 0.92	4.42 ± 1.28	3.48 ± 0.67	3.31 ± 0.69	3.33 ± 0.93	**< 0.001**	0.422	0.082	0.540	0.737	0.943	0.857
	12	5.44 ± 1.14	4.86 ± 1.31	4.95 ± 1.10	3.50 ± 0.69	3.75 ± 0.87	3.82 ± 1.01							
	24	5.05 ± 2.03	4.82 ± 0.89	4.34 ± 0.91	3.33 ± 0.84	3.00 ± 0.53	3.04 ± 0.44							
**Isometric tetanic force (mN)**	6	1028 ± 124	984 ± 104	905 ± 113	815 ± 120	911 ± 282	814 ± 141	0.632	0.950	**< 0.001**	**0.507**	**0.008**	0.719	0.596
	12	1303 ± 140	1349 ± 153	1273 ± 201	1355 ± 227	1346 ± 307	1479 ± 216							
	24	1332 ± 182	1278 ± 184	1341 ± 197	1396 ± 194	1416 ± 257	1398 ± 216							
**Specific isometric tetanic force (N/cm^2)**	6	21.2 ± 2.86	20.2 ± 1.98	19.08 ± 2.72	14.0 ± 2.22	14.8 ± 2.70	15.3 ± 2.71	**< 0.001**	0.457	**< 0.001**	0.202	0.201	0.681	0.628
	12	23.8 ± 2.89	23.7 ± 2.86	22.5 ± 3.50	15.9 ± 2.14	15.4 ± 2.87	17.3 ± 2.47							
	24	20.7 ± 3.96	18.6 ± 2.67	20.5 ± 2.66	13.0 ± 2.45	11.7 ± 2.30	12.9 ± 2.22							
**Twitch : Tetanic**	6	0.23 ± 0.04	0.24 ± 0.04	0.23 ± 0.05	0.25 ± 0.04	0.22 ± 0.07	0.21 ± 0.03	0.370	0.330	0.066	0.895	0.728	0.826	0.370
	12	0.23 ± 0.05	0.20 ± 0.05	0.21 ± 0.07	0.22 ± 0.05	0.24 ± 0.04	0.22 ± 0.04							
	24	0.24 ± 0.06	0.26 ± 0.06	0.22 ± 0.04	0.26 ± 0.07	0.25 ± 0.04	0.24 ± 0.06							

**Isometric twitch time to peak (ms)**	6	59 ± 2.17	56.2 ± 12.0	73.3 ± 32.2	55.1 ± 18.6	105 ± 79.4	69.9 ± 30.0	0.211	0.991	0.736	0.397	0.686	**0.053**	0.239
	12	72 ± 36.6	69.5 ± 23.6	55.7 ± 1.03	78.1 ± 50.8	64.7 ± 16.9	60.3 ± 11.7							
	24	67.0 ± 34.8	55.4 ± 1.43	83.4 ± 58.7	82.4 ± 44.5	64.8 ± 16.1	77.8 ± 12.1							
**Isometric twitch 1/2 relaxation time (ms)**	6	20.7 ± 5.92	21.6 ± 5.33	20.6 ± 7.57	22.9 ± 4.26	20.6 ± 6.12	22.3 ± 7.29	0.596	0.889	**< 0.001**	0.587	0.328	0.966	0.855
	12	16.0 ± 1.51	15.7 ± 2.16	15.4 ± 1.31	14.3 ± 1.06	14.3 ± 2.01	15.4 ± 1.50							
	24	14.8 ± 1.21	15.0 ± 1.34	14.1 ± 1.07	13.9 ± 1.52	13.8 ± 0.61	13.2 ± 0.52							
**Isometric tetanic rate of contraction (N/s)**	6	24.6 ± 5.42	24.1 ± 5.63	22.8 ± 5.22	19.9 ± 5.72	21.4 ± 8.35	20.1 ± 4.10	0.444	0.882	**< 0.001**	0.817	0.079	0.797	0.958
	12	34.8 ± 7.49	37.0 ± 9.94	34.8 ± 7.09	38.0 ± 12.7	38.0 ± 10.4	40.5 ± 10.5							
	24	40.6 ± 7.29	36.3 ± 7.26	36.5 ± 8.53	42.1 ± 6.26	40.7 ± 10.6	40.1 ± 8.54							
**Isometric tetanic rate of relaxation (N/s)**	6	22.1 ± 7.16	19.3 ± 5.74	20.2 ± 7.39	19.4 ± 11.0	21.4 ± 9.71	17.8 ± 6.28	**0.018**	0.974	**< 0.001**	0.894	0.119	0.905	0.770
	12	30.8 ± 10.1	33.8 ± 14.3	34.0 ± 9.36	40.8 ± 11.7	37.9 ± 15.1	44.0 ± 10.1							
	24	29.1 ± 6.41	28.3 ± 8.91	31.3 ± 15.6	37.9 ± 15.3	37.1 ± 17.2	33.3 ± 13.0							
**Maximal eccentric force (mN)**	6	1991 ± 199	2084 ± 136	1960 ± 282	1975 ± 202	1958 ± 387	1858 ± 144	0.628	0.945	**< 0.001**	0.992	0.450	0.363	0.738
	12	2407 ± 256	2442 ± 253	2490 ± 185	2279 ± 303	2484 ± 503	2589 ± 203							
	24	2287 ± 393	2319 ± 348	2256 ± 463	2452 ± 338	2436 ± 594	2241 ± 383							
**Specific eccentric force (N/cm2)**	6	40.9 ± 2.88	43.0 ± 3.97	41.2 ± 6.02	33.8 ± 6.16	32.6 ± 3.55	35.5 ± 8.23	**< 0.001**	0.719	**< 0.001**	0.746	**0.002**	0.689	0.721
	12	44.0 ± 5.86	43.0 ± 5.22	44.0 ± 2.55	26.9 ± 3.17	28.5 ± 4.20	30.5 ± 5.00							
	24	35.4 ± 7.66	33.7 ± 4.44	34.5 ± 7.22	22.8 ± 4.60	20.3 ± 5.36	20.6 ± 3.96							
**Tetanic force (% of initial post ECC)**	6	62.6 ± 16.5	64.7 ±12.1	63.7 ± 14.1	39.3 ± 10.7	37.6 ± 7.45	47.8 ± 7.58	**< 0.001**	0.205	0.100	0.650	**0.016**	0.517	0.088
	12	57.2 ± 8.50	67.1 ± 5.40	76.7 ± 15.3	31.2 ± 10.2	33.7 ± 17.0	19.8 ± 4.80							
	24	72.9 ± 14.0	69.7 ± 21.5	76.8 ± 4.91	34.2 ± 19.7	28.4 ± 11.4	45.8 ± 23.9							
**Isometric tetanic rate of contraction (% of initial post ECC)**	6	55.0 ± 16.1	56.8 ± 13.4	56.5 ± 23.1	43.1 ± 14.6	41.4 ± 16.1	47.1 ± 5.96	**< 0.001**	0.150	**< 0.001**	0.576	**0.050**	0.137	0.649
	12	55.2 ± 11.4	61.5 ± 14.3	68.1 ± 13.3	36.3 ± 12.2	38.0 ± 20.1	23.8 ± 6.84							
	24	73.6 ± 17.5	71.8 ± 19.7	94.3 ± 26.4	46.4 ± 27.4	39.0 ± 17.7	64.1 ± 34.7							

**Isometric Tetanic t rate of relaxation (% of initial post ECC)**	6	76.8 ± 24.5	75.1 ± 11.2	70.0 ± 9.82	46.8 ± 12.1	41.0 ± 5.17	54.0 ± 5.00	**< 0.001**	0.936	0.423	0.920	**< 0.001**	0.703	0.390
	12	74.2 ± 9.83	84.2 ± 11.7	86.7 ± 18.4	31.9 ± 8.80	35.5 ± 22.7	18.0 ± 3.74							
	24	96.1 ± 23.8	91.5 ± 31.1	97.5 ± 48.1	24.2 ± 20.0	20.1 ± 9.23	31.3 ± 13.5							

Table S7. Bone microarchitectural parameters, from male wildtype and *mdx* offspring born to non-stressed or stressed *mdx*-heterozygous mothers at 6, 12 and 24 weeks of age. Values are mean ± SD. Three different stress paradigms were employed including a non-stressed condition (NS), a 30-second scruff restraint (SR), and a 30-minute tube restraint (TR).

**Parameter**	**Age (weeks)**	**Wildtype**			***mdx***			**Genotype** (*p* value)	**Paradigm** (*p* value)	**Time** (*p* value)	**Genotype x Paradigm** (*p* value)	**Genotype x Time** (*p* value)	**Paradigm x Time** (*p* value)	**Genotype x Paradigm x Time** (*p* value)
		NS	SR	TR	NS	SR	TR							
**Femur length (mm)**	6	11.2 ± 0.25	11.4 ± 0.25	11.1 ± 0.34	10.8 ± 0.47	10.8 ± 0.41	10.8 ± 0.27	**0.685**	**0.044**	**< 0.001**	0.959	**< 0.001**	0.596	0.500
	12	12.7 ± 0.16	12.7 ± 0.22	12.7 ± 0.10	12.7 ± 0.34	12.8 ± 0.48	12.7 ± 0.25							
	24	13.0 ± 0.15	13.2 ± 0.22	13.2 ± 0.27	13.4 ± 0.21	13.6 ± 0.19	13.4 ± 0.10							
**Trabecular volume ratio (% of tissue volume)**	6	10.4 ± 3.24	10.6 ± 3.01	9.55 ± 2.08	6.41 ± 2.24	5.40 ± 1.96	4.15 ± 0.76	**< 0.001**	0.600	**< 0.001**	0.250	**< 0.001**	0.526	0.976
	12	7.13 ± 3.50	8.18 ± 2.54	8.49 ± 0.77	5.05 ± 4.04	4.27 ± 1.78	4.90 ± 2.03							
	24	4.03 ± 1.70	4.65 ± 1.84	3.62 ± 0.78	4.24 ± 0.89	3.27 ± 0.98	3.53 ± 1.47							
**Trabecular number (1/mm)**	6	1.67 ± 0.47	1.69 ± 0.43	1.57 ± 0.30	1.12 ± 0.36	0.96 ± 0.32	0.75 ± 0.12	**< 0.001**	0.557	**< 0.001**	0.251	**< 0.001**	0.626	0.948
	12	1.19 ± 0.57	1.34 ± 0.39	1.37 ± 0.14	0.91 ± 0.66	0.76 ± 0.28	0.88 ± 0.35							
	24	0.72 ± 0.31	0.79 ± 0.27	0.63 ± 0.14	0.77 ± 0.17	0.61 ± 0.15	0.66 ± 0.23							
**Trabecular thickness (μm)**	6	62.1 ± 3.90	62.3 ± 3.51	60.5 ± 2.36	56.6 ± 3.33	55.4 ± 3.55	55.0 ± 1.65	**< 0.001**	0.951	**0.002**	0.487	0.222	0.285	0.897
	12	58.7 ± 6.06	60.6 ± 3.30	62.2 ± 3.04	53.2 ± 4.93	54.6 ± 6.53	55.3 ± 1.97							
	24	56.6 ± 4.94	57.9 ± 4.42	57.4 ± 2.51	55.5 ± 2.06	53.3 ± 5.64	52.9 ± 3.82							
**Trabecular separation (μm)**	6	377 ± 91.2	357 ± 52.4	382 ± 59.1	428 ± 98.0	482 ± 117	561 ± 92.0	**< 0.001**	0.083	**< 0.001**	0.135	**< 0.001**	0.351	0.404
	12	336 ± 70.9	325 ± 47.4	328 ± 41.3	358 ±76.0	373 ± 46.1	369 ± 54.8							
	24	335 ±50.7	337 ± 31.3	358 ± 29.8	325 ± 22.9	351 ± 38.5	349.2 ± 24.4							
**Cortical thickness (μm)**	6	183 ± 12.2	183 ± 18.8	181 ± 10.8	164 ± 17.1	164 ± 14.2	160 ± 7.95	**< 0.001**	0.363	**< 0.001**	0.961	**0.003**	0.904	0.926
	12	220 ± 11.0	217 ± 16.2	218 ± 5.82	215 ± 16.6	218 ± 14.9	214 ± 10.7							
	24	222 ± 7.41	229 ± 6.49	220 ± 9.35	218 ± 4.51	220 ± 9.46	215 ± 6.09							

	6	1.53 ± 0.16	1.56 ± 0.12	1.45 ± 0.07	1.37 ± 0.21	1.38 ± 0.10	1.34 ± 0.10							
**Marrow area (mm^2)**	12	1.43 ± 0.11	1.39 ± 0.17	1.54 ± 0.15	1.33 ± 0.21	1.48 ± 0.24	1.42 ± 0.15	**0.006**	0.148	**0.002**	0.715	0.203	0.194	0.443
	24	1.47 ± 0.15	1.58 ± 0.21	1.65 ± 0.09	1.46 ± 0.14	1.52 ± 0.23	1.58 ± 0.15							
	6	0.84 ± 0.09	0.85 ± 0.12	0.81 ± 0.07	0.71 ± 0.13	0.71 ± 0.09	0.67 ± 0.05							
**Cortical area (mm^2)**	12	1.02 ± 0.09	1.04 ± 0.70	1.04 ± 0.05	0.99 ± 0.13	1.04 ± 0.14	1.00 ± 0.09	**< 0.001**	0.230	**< 0.001**	0.839	**0.009**	0.723	0.956
	24	1.06 ± 0.08	1.12 ± 0.07	1.10 ± 0.04	1.04 ± 0.04	1.07 ± 0.10	1.04 ± 0.07							
	6	5.94 ± 0.28	5.95 ± 0.27	5.87 ± 0.28	5.52 ± 0.48	5.50 ± 0.23	5.40 ± 0.21’							
**Periosteal perimeter (mm)**	12	5.99 ± 0.24	5.99 ± 0.29	6.16 ± 0.23	5.81 ± 0.44	6.09 ± 0.46	5.99 ± 0.27	**< 0.001**	0.228	**< 0.001**	0.683	**0.008**	0.376	0.776
	24	6.11 ± 0.29	6.31 ± 0.33	6.41 ± 0.90	6.13 ± 0.21	6.23 ± 0.40	6.26 ± 0.27							
	6	4.86 ± 0.28	4.90 ± 0.25	4.76 ± 0.25	4.53 ± 0.38	4.63 ± 0.19	4.51 ± 0.19							
**Endocortical perimeter (mm)**	12	4.75 ± 0.22	4.82 ± 0.37	5.00 ± 0.23	4.66 ± 0.43	4.87 ± 0.41	4.79 ± 0.31	**0.011**	**0.051**	**< 0.001**	**0.263**	0.177	0.263	0.820
	24	4.84 ± 0.26	5.05 ± 0.41	5.27 ± 0.25	4.90 ± 0.24	4.99 ± 0.41	5.09 ± 0.28							
	6	0.54 ± 0.10	0.55 ± 0.12	0.49 ± 0.06	0.41 ± 0.13	0.40 ± 0.08	0.37 ± 0.06							
**Mean polar moment of inertia (mm^4)**	12	0.65 ± 0.10	0.66 ± 0.11	0.70 ± 0.09	0.58 ± 0.16	0.69 ± 0.17	0.64 ± 0.10	**< 0.001**	0.177	**< 0.001**	0.824	0.070	0.363	0.746
	24	0.70 ± 0.12	0.79 ± 0.14	0.80 ± 0.05	0.69 ± 0.08	0.74 ± 0.16	0.73 ± 0.11							

Table S8. Bone mechanical parameters from male wildtype and *mdx* offspring born to non-stressed or stressed *mdx*-heterozygous mothers at 24 weeks of age. Values are mean ± SD. Three different stress paradigms were employed including a non-stressed condition (NS), a 30-second scruff restraint (SR), and a 30-minute tube restraint (TR). CC, cranio-caudal; ML, medial–latera.

Parameter	Age (weeks)	Wildtype			*mdx*			Genotype (*p* value)	Paradigm (*p* value)	Genotype x Paradigm (*p* value)
		NS	SR	TR	NS	SR	TR			
**Diameter ML (mm)**	24	2.15 ± 0.09	2.21 ± 0.11	2.22 ± 0.05	2.21 ± 0.09	2.27 ± 0.15	2.25 ± 0.06	0.141	0.181	0.880
**Diameter CC (mm)**	24	1.59 ± 0.08	1.66 ± 0.07	1.63 ± 0.04	1.55 ± 0.06	1.57 ± 0.08	1.59 ± 0.06	**0.007**	0.153	0.629
**Ultimate strain (%)**	24	5.20 ± 1.23	5.02 ± 1.10	4.81 ± 1.06	5.52 ± 1.54	6.03 ± 1.70	5.06 ± 1.15	0.200	0.529	0.706
**Yield displacement (mm)**	24	0.21 ± 0.04	0.20 ± 0.04	0.21 ± 0.04	0.27 ± 0.07	0.25 ± 0.03	0.24 ± 0.04	**0.003**	0.660	0.734
**Yield strain (%)**	24	3.15 ± 0.60	3.12 ± 0.63	3.26 ± 0.50	3.90 ± 1.02	3.63 ± 0.27	3.64 ± 0.72	**0.015**	0.821	0.761
**Post yield strain (%)**	24	4.13 ± 3.72	4.75 ± 3.54	4.31 ± 4.60	5.43 ± 6.24	8.19 ± 7.79	2.57 ± 2.19	0.506	0.310	0.423
**Failure deformation (mm)**	24	0.48 ± 0.22	0.51 ± 0.23	0.49 ± 0.28	0.65 ± 0.45	0.78 ± 0.52	0.42 ± 0.19	0.239	0.358	0.406
**Failure strain (%)**	24	7.28 ± 3.70	7.87 ± 3.40	7.56 ± 4.39	9.34 ± 6.38	11.8 ± 7.94	6.21 ± 2.87	0.312	0.342	0.411
**Failure energy (J)**	24	0.006 ± 0.004	0.007 ± 0.005	0.006 ± 0.004	0.008 ± 0.006	0.009 ± 0.006	0.005 ± 0.004	0.646	0.428	0.659
**Toughness (J/mm3)**	24	0.0009 ± 0.0004	0.001 ± 0.0008	0.0008 ± 0.0006	0.001 ± 0.0009	0.001 ± 0.0007	0.0006 ± 0.0004	0.853	0.502	0.654
**Yield force (N)**	24	14.3 ± 3.79	15.4 ± 3.19	17.4 ± 4.28	15.2 ± 2.36	15.1 ± 2.37	15.1 ± 3.07	0.600	0.470	0.452
**Yield stress (MPa)**	24	13.8 ± 4.30	13.6 ± 4.25	15.3 ± 4.22	14.9 ± 2.09	14.7 ± 4.42	13.5 ± 1.77	0.896	0.983	0.554
**Stiffness (N/mm)**	24	81.3 ± 13.7	98.5 ± 12.0	91.7 ± 9.86	76.5 ± 17.9	81.1 ± 19.2	74.9 ± 18.7	**0.009**	0.143	0.417
**Post yield displacement (mm)**	24	0.27 ± 0.23	0.31 ± 0.24	0.28 ± 0.30	0.38 ± 0.44	0.54 ± 0.51	0.17 ± 0.14	0.449	0.328	0.435
**Ultimate force (N)**	24	18.3 ± 2.18	19.8 ± 2.96	20.6 ± 2.43	18.0 ± 3.51	18.8 ± 3.34	17.6 ± 3.37	0.137	0.511	0.474
**Elastic modulus ((MPa)**	24	512 ± 111	558 ± 536	525 ± 65.3	523 ± 166	537 ± 183	455 ± 114	0.542	0.595	0.748
**Ultimate stress (MPa)**	24	17.8 ± 4.15	17.3 ± 4.57	18.01 ± 2.57	17.7 ± 3.66	17.8 ± 3.08	15.6 ± 1.78	0.573	0.805	0.613
**Ultimate displacement (mm)**	24	0.34 ± 0.07	0.32 ± 0.07	0.31 ± 0.06	0.38 ± 0.09	0.41 ± 0.10	0.34 ± 0.07	**0.040**	0.436	0.590
**Failure force (N)**	24	16.5 ± 2.39	17.1 ± 3.69	18.7 ± 4.51	14.7 ± 5.56	13.4 ± 7.52	15.3 ± 3.40	**0.042**	0.596	0.789
**Failure stress (MPa)**	24	16.1 ± 4.49	14.7 ± 3.42	16.3 ± 3.90	14.1 ± 4.40	11.7 ± 6.46	13.9 ± 3.30	0.074	0.440	0.958

Table S9. The mass of organs from male wildtype and *mdx* mice born to non-stressed or stressed *mdx*-heterozygous mothers at 6, 12 and 24 weeks of age. Values are mean ± SD. Three different stress paradigms were employed including a non-stressed condition, and a stressed condition which combined data from the 30-second scruff restraint and 30-minute tube restraint paradigms.

**Parameter**	**Age (weeks)**	**Wildtype**		***mdx***		**Genotype** (*p* value)	**Paradigm** (*p* value)	**Time** (*p* value)	**Genotype x Paradigm** (*p* value)	**Genotype x Time** (*p* value)	**Paradigm x Time** (*p* value)	**Genotype x Paradigm x Time** (*p* value)
		Non-stressed	Stressed	Non-stressed	Stressed							
**Adrenal mass (mg)**	6	2.26 ± 0.29	2.20 ± 0.24	2.56 ± 0.38	2.40 ± 0.24	**< 0.001**	0.979	0.199	0.621	0.447	0.214	0.841
	12	2.19 ± 0.27	2.30 ± 0.30	2.61 ± 0.20	2.66 ± 0.33							
	24	2.29 ± 0.19	2.31 ± 0.26	2.56 ± 0.26	2.61 ± 0.26							
**Adrenal mass (% of body mass)**	6	0.010 ± 0.001	0.010 ± 0.001	0.012 ± 0.001	0.011 ± 0.001	**< 0.001**	0.534	**< 0.001**	0.237	0.088	0.527	0.695
	12	0.007 ± 0.001	0.008 ± 0.001	0.009 ± 0.001	0.008 ± 0.001							
	24	0.006 ± 0.001	0.005 ± 0.001	0.007 ± 0.001	0.007 ± 0.001							
**Heart mass (mg)**	6	121 ± 11.3	116 ± 10.7	109 ± 14.6	107 ± 11.9	0.433	0.296	**< 0.001**	0.513	**0.021**	0.187	0.951
	12	140 ± 14.1	143 ± 14.0	137 ± 19.8	144 ± 24.7							
	24	159 ± 11.8	165 ± 11.5	164 ± 11.2	171 ± 12.8							
**Heart mass (% of body mass)**	6	0.527 ± 0.029	0.516 ± 0.031	0.517 ± 0.028	0.506 ± 0.035	0.193	0.992	**< 0.001**	0.818	**< 0.001**	0.273	0.933
	12	0.453 ± 0.038	0.468 ± 0.040	0.442 ± 0.027	0.448 ± 0.032							
	24	0.382 ± 0.034	0.382 ± 0.020	0.429 ± 0.029	0.448 ± 0.032							
**Brain mass (mg)**	6	438 ± 8.28	428 ± 17.1	414 ± 21.9	415 ± 16.1	**0.005**	0.246	**< 0.001**	0.294	**0.017**	0.071	0.252
	12	429 ± 18.2	436 ± 13.5	433 ± 24.2	433 ± 21.7							
	24	449 ± 6.72	453 ± 11.6	437 ± 13.6	454 ± 15.0							
**Brain mass (% of body mass)**	6	1.916 ± 0.152	1.910 ± 0.134	1.992 ± 0.236	1.981 ± 0.166	**0.057**	0.738	**< 0.001**	0.580	0.840	0.948	0.517
	12	1.388 ± 0.099	1.435 ± 0.0745	1.406 ± 0.962	1.363 ± 0.119							
	24	1.081 ± 0.068	1.054 ± 0.076	1.144 ± 0.071	1.142 ± 0.121							

Table S10. Open-field physical activity (10 min assay) of male wildtype and *mdx* offspring born to non-stressed or stressed *mdx*-heterozygous mothers at 6, 12 and 24 weeks of age. Values are mean ± SD. Three different stress paradigms were employed including a non-stressed condition, and a stressed condition which combined data from the 30-second scruff restraint and 30-minute tube restraint paradigms.

**Parameter**	**Age (weeks)**	**Wildtype**		***mdx***		**Genotype** (*p* value)	**Paradigm** (*p* value)	**Time** (*p* value)	**Genotype x Paradigm** (*p* value)	**Genotype x Time** (*p* value)	**Paradigm x Time** (*p* value)	**Genotype x Paradigm x Time** (*p* value)
		Non-stressed	Stressed	Non-stressed	Stressed							
**Total distance (m)**	6	4.54 ± 1.30	4.32 ± 1.22	4.28 ± 0.99	3.23 ± 1.23	0.976	0.243	**< 0.001**	**0.001**	0.060	0.407	0.546
	12	3.04 ± 1.27	3.62 ± 1.39	3.85 ± 1.12	3.12 ± 0.99							
	24	1.95 ± 1.03	2.90 ± 1.35	3.43 ± 1.04	2.43 ± 1.26							
**Movement time (s)**	6	334 ± 55.5	305 ± 46.7	273 ± 48.8	203 ± 70.4	**0.001**	**0.017**	**0.034**	**< 0.001**	**0.013**	0.097	0.259
	12	305 ± 45.5	342 ± 76.6	316 ± 59.0	292 ± 80.3							
	24	284 ± 48.1	311 ± 79.3	339 ± 62.2	241 ± 55.9							
**Vertical activity (counts)**	6	80.5 ± 39.2	70.9 ± 36.2	60.5 ± 47.4	47.2 ± 35.8	0.488	0.749	**< 0.001**	**0.001**	0.653	0.658	0.078
	12	106 ± 55.3	163 ± 73.4	151 ± 78.5	116 ± 103							
	24	74.2 ± 37.6	125 ± 97.6	135 ± 45.9	64.7 ± 39.9							
**Ambulatory episode average velocity (m/s)**	6	0.062 ± 0.008	0.069 ± 0.221	0.056 ± 0.009	0.054 ± 0.008	**< 0.001**	0.882	**0.040**	**0.003**	0.543	0.763	0.485
	12	0.062 ± 0.010	0.066 ± 0.009	0.055 ± 0.006	0.052 ± 0.006							
	24	0.055 ± 0.009	0.062 ± 0.007	0.056 ± 0.008	0.047 ± 0.005							
**Corner time (legacy; s)**	6	34.9 ± 14.2	29.7 ± 14.9	57.6 ± 15.9	60.39 ± 28.4	**< 0.001**	0.736	**< 0.001**	0.116	**< 0.001**	0.474	0.982
	12	20.8 ± 13.3	14.8 ± 7.31	22.0 ± 10.8	25.0 ± 22.0							
	24	7.48 ± 6.58	9.30 ± 7.38	9.91 ± 4.83	18.5 ± 11.8							
**Margin time (legacy; s)**	6	434 ± 78.4	399 ± 82.3	515 ± 53.7	474 ± 133	**< 0.001**	0.860	**< 0.001**	0.170	0.678	0.144	0.527
	12	337 ± 61.5	301 ± 60.0	357 ± 80.4	381 ± 105							
	24	229 ± 121	228 ± 106	273 ± 51.1	344 ± 86.0							

**Centre time (legacy; s)**	6	166 ± 78.4	182 ± 73.0	85.2 ± 53.7	56.7 ± 42.4	**< 0.001**	0.379	**< 0.001**	**0.032**	0.285	0.443	0.916
	12	263 ± 61.5	299 ± 51.0	243 ± 80.5	219 ± 105							
	24	371 ± 121	372 ± 106	327 ± 51.1	256 ± 86.0							

Table S11. Open field physical activity of male wildtype and *mdx* offspring born to non-stressed or stressed *mdx*-heterozygous mothers in response to a 30-second scruff restraint at 6, 12 and 24 weeks of age. Values are mean ± SD. Three different stress paradigms were employed including a non-stressed condition, and a stressed condition which combined data from the 30-second scruff restraint and 30-minute tube restraint paradigms.

**Parameter**	**Age (weeks)**	**Wildtype**		***mdx***		**Genotype** (*p* value)	**Paradigm** (*p* value)	**Time** (*p* value)	**Genotype x Paradigm** (*p* value)	**Genotype x Time** (*p* value)	**Paradigm x Time** (*p* value)	**Genotype x Paradigm x Time** (*p* value)
		Non-stressed	Stressed	Non-stressed	Stressed							
	6	40.4 ± 18.0	62.1 ± 33.7	13.4 ± 11.5	11.7 ± 13.7							
**Total distance (% of intervention)**	12	61.0 ± 21.4	54.9 ± 21.7	3.93 ± 2.45	6.63 ± 7.10	< **0.001**	0.104	**0.023**	0.069	**0.003**	**0.004**	**0.002**
	24	135 ± 119	54.8 ± 26.9	4.10 ± 3.63	6.57 ± 6.83							
	6	58.1 ± 10.9	75.2 ± 22.9	21.8 ± 14.3	22.5 ± 23.6							
**Movement time (% of intervention)**	12	78.4 ± 11.2	73.2 ± 13.9	7.93 ± 5.09	10.0 ± 7.02	< **0.001**	0.526	0.413	0.987	**0.001**	0.234	0.141
	24	76.4 ± 22.4	70.1 ± 26.8	4.59 ± 2.27	7.47 ± 8.43							
	6	40.9 ± 11.3	59.7 ± 36.5	7.84 ± 9.78	3.72 ± 9.13							
**Vertical activity (% of intervention)**	12	83.1 ± 103	41.4 ± 23.2	0.05 ± 0.13	1.61 ± 4.25	< **0.001**	0.286	0.900	0.350	0.492	0.205	0.099
	24	66.4 ± 59.0	51.4 ± 36.8	0.0 ± 0.0	0.06 ± 021							
	6	79.2 ± 11.3	77.5 ± 18.2	57.6 ± 31.6	63.1 ± 13.4							
**Ambulatory episode average velocity (% of intervention)**	12	85.7 ± 17.7	80.3 ± 14.7	69.2 ± 18.2	65.6 ± 36.7	< **0.001**	0.313	0.376	0.658	**0.087**	0.503	0.910
	24	92.1 ± 11.2	84.1 ± 10.7	58.9 ± 15.4	51.1 ± 28.9							
	6	196 ± 134	192 ± 75.1	146 ± 55.1	241 ± 143							
**Corner time (legacy; % of intervention)**	12	185 ± 187	197 ± 172	478 ± 298	614 ± 1014	0.258	0.473	**0.014**	0.761	0.720	0.829	0.722
	24	421 ± 542	1048 ± 2767	1069 ± 1066	1097 ± 2083							
	6	111 ± 21.9	113 ± 31.4	113 ± 14.0	141 ± 57.9							
**Margin time (legacy; % of intervention)**	12	116 ± 18.9	128 ± 34.9	159 ± 70.1	170 ± 53.8	**0.030**	0.332	**< 0.001**	0.257	0.682	0.991	0.072
	24	142 ± 63.4	210 ± 182	226 ± 50.3	181 ± 40.7							

**Center time (legacy; % of intervention)**	6	83.1 ± 49.7	111 ± 62.0	28.4 ± 47.1	3.47 ± 6.39	**< 0.001**	0.619	**0.045**	0.356	0.967	0.766	**0.032**
	12	81.2 ± 23.8	77.8 ± 29.8	1.81 ± 3.01	1.98 ± 3.28							
	24	89.8 ± 28.7	72.7 ± 39.1	2.29 ± 6.51	2.42 ± 3.45							

Table S12. Physiological parameters of heart function from wildtype and *mdx* offspring born to non-stressed or stressed mdx-*heterozygous* mothers at 6, 12 and 24 weeks of age. Values are mean ± SD. MAP = Mean arterial blood pressure. Shock index is calculated by maximum heart rate/low systolic blood pressure. Three different stress paradigms were employed including a non-stressed condition, and a stressed condition which combined data from the 30-second scruff restraint and 30-minute tube restraint paradigms.

**Parameter**	**Age (weeks)**	**Wildtype**		***mdx***		**Genotype** (*p* value)	**Paradigm** (*p* value)	**Time** (*p* value)	**Genotype x Paradigm** (*p* value)	**Genotype x Time** (*p* value)	**Paradigm x Time** (*p* value)	**Genotype x Paradigm x Time** (*p* value)
		Non-stressed	Stressed	Non-stressed	Stressed							
**MAP (mmHg)**	6	144 ± 23.7	137 ± 24.0	113 ± 29.1	126 ± 31.5	**< 0.001**	**0.037**	0.715	0.150	**0.035**	0.604	0.395
	12	146 ± 23.2	149 ± 22.7	105 ± 16.9	130 ± 25.6							
	24	146 ± 26.0	158 ± 21.0	100 ± 27.9	108 ± 27.5							
**Heart rate (bpm)**	6	622 ± 40.9	636 ± 63.9	650 ± 38.3	617 ± 40.6	0.270	**0.046**	**< 0.001**	**0.014**	0.771	0.546	0.973
	12	662 ± 75.1	653 ± 42.9	702 ± 62.6	649 ± 47.5							
	24	667 ± 28.5	674 ± 42.9	692 ± 81.6	661 ± 41.4							
**Shock index**	6	4.81 ± 1.19	5.08 ± 1.21	6.41 ± 1.81	5.29 ± 1.64	**< 0.001**	**0.001**	0.685	0.159	0.796	0.558	0.402
	12	5.21 ± 1.71	4.76 ± 1.06	6.64 ± 1.20	5.39 ± 1.43							
	24	5.56 ± 1.43	4.45 ± 0.55	6.76 ± 2.09	5.82 ± 1.45							

Table S13. The mass of muscle and bone from male wildtype and *mdx* mice born to non-stressed or stressed *mdx*-heterozygous mothers at 6, 12 and 24 weeks of age. Values are mean ± SD. Three different stress paradigms were employed including a non-stressed condition, and a stressed condition which combined data from the 30-second scruff restraint and 30-minute tube restraint paradigms.

**Parameter**	**Age (weeks)**	**Wildtype**		***mdx***		**Genotype** (*p* value)	**Paradigm** (*p* value)	**Time** (*p* value)	**Genotype x Paradigm** (*p* value)	**Genotype x Time** (*p* value)	**Paradigm x Time** (*p* value)	**Genotype x Paradigm x Time** (*p* value)
		Non-stressed	Stressed	Non-stressed	Stressed							
**Tibialis anterior mass (mg)**	6	37.3 ± 6.40	35.7 ± 4.59	42.2 ± 9.22	41.0 ± 9.60	**< 0.001**	0.327	**< 0.001**	0.827	**< 0.001**	0.196	0.990
	12	42.9 ± 3.42	43.9 ± 2.80	67.5 ± 8.65	68.6 ± 10.8							
	24	49.4 ± 2.52	52.8 ± 4.27	87.1 ± 5.01	91.4 ± 10.9							
**Tibialis anterior mass (% of body mass)**	6	0.162 ± 0.022	0.159 ± 0.014	0.198 ± 0.017	0.192 ± 0.028	**< 0.001**	0.733	0.427	0.360	**< 0.001**	0.589	0.855
	12	0.139 ± 0.016	0.144 ± 0.007	0.218 ± 0.018	0.214 ± 0.015							
	24	0.119 ± 0.010	0.122 ± 0.008	0.228 ± 0.018	0.229 ± 0.022							
**Gastrocnemius mass (mg)**	6	127 ± 14.2	127 ± 13.6	123 ± 22.5	119 ± 19.3	**< 0.001**	0.172	**< 0.001**	0.777	**< 0.001**	0.361	0.919
	12	153 ± 14.1	163 ± 8.43	185 ± 34.9	191 ± 30.8							
	24	181 ± 8.82	187 ± 10.5	202 ± 15.0	209 ± 18.2							
**Gastrocnemius mass (% of body mass)**	6	0.551 ± 0.032	0.562 ± 0.026	0.579 ± 0.030	0.561 ± 0.041	**< 0.001**	0.399	**< 0.001**	0.057	**< 0.001**	0.182	0.356
	12	0.495 ± 0.038	0.537 ± 0.015	0.594 ± 0.053	0.594 ± 0.045							
	24	0.435 ± 0.024	0.433 ± 0.018	0.528 ± 0.036	0.525 ± 0.059							
**Soleus mass (mg)**	6	6.31 ± 0.77	6.28 ± 0.82	7.30 ± 1.54	7.11 ± 1.27	**< 0.001**	0.682	**< 0.001**	0.950	**< 0.001**	0.106	0.974
	12	8.40 ± 1.19	8.01 ± 1.10	11.9 ± 1.46	11.6 ± 2.24							
	24	10.7 ± 1.17	11.5 ± 10.5	15.0 ± 1.21	15.7 ± 1.16							
**Soleus mass (% of body mass)**	6	0.027 ± 0.003	0.028 ± 0.003	0.034 ± 0.005	0.034 ± 0.004	**< 0.001**	0.453	**0.032**	0.309	**< 0.001**	0.332	0.880
	12	0.027 ± 0.003	0.026 ± 0.003	0.038 ± 0.004	0.036 ± 0.004							
	24	0.026 ± 0.003	0.027 ± 0.002	0.039 ± 0.005	0.040 ± 0.005							

**Tibia mass (mg)**	6	44.2 ± 4.52	46.4 ±5.94	40.9 ± 7.94	39.6 ± 5.00	**0.008**	**0.025**	**< 0.001**	0.893	0.331	0.062	0.555
	12	54.1 ± 4.93	53.8 ± 6.34	51.3 ± 7.45	53.0 ± 9.47							
	24	66.0 ± 3.81	71.6 ± 5.87	64.1 ± 3.89	70.3 ± 5.77							
**Tibia mass (% of body mass)**	6	0.190 ± 0.012	0.206 ± 0.021	0.194 ± 0.028	0.189 ± 0.023	0.325	0.171	**< 0.001**	0.231	**0.017**	0.551	0.260
	12	0.176 ± 0.026	0.177 ± 0.015	0.165 ± 0.014	0.164 ± 0.014							
	24	0.160 ± 0.013	0.166 ± 0.134	0.168 ± 0.013	0.176 ± 0.014							
**Femur mass (mg)**	6	55.3 ± 6.00	54.9 ± 7.13	45.9 ± 9.22	44.8 ± 5.82	< **0.001**	0.172	**< 0.001**	0.780	**0.006**	0.366	0.763
	12	68.5 ± 4.17	70.1 ± 6.65	64.9 ± 10.1	68.1 ± 9.58							
	24	90 ± 7.43	94.7 ± 6.00	91.0 ± 3.40	92.9 ± 7.79							
**Femur mass (% of body mass)**	6	0.240 ± 0.016	0.240 ± 0.016	0.216 ± 0.019	0.213 ± 0.022	**0.003**	0.575	**0.010**	0.223	**< 0.001**	0.574	0.963
	12	0.221 ± 0.018	0.221 ± 0.018	0.209 ± 0.014	0.212 ± 0.011							
	24	0.216 ± 0.017	0.216 ± 0.172	0.238 ± 0.011	0.232 ± 0.019							
**Tibia length (mm)**	6	15.8 ± 0.36	16.0 ± 0.53	15.8 ± 0.97	15.6 ± 0.52	0.627	0.154	**< 0.001**	0.645	0.427	0.224	0.145
	12	17.4 ± 0.47	17.2 ± 0.51	17.2 ± 0.67	17.4 ± 0.56							
	24	18.5 ± 0.46	18.8 ± 0.62	18.5 ± 0.75	19.0 ± 0.71							
**Femur length (mm)**	6	13.5 ± 0.60	13.4 ± 1.02	12.8 ± 1.03	12.8 ± 0.89	0.625	0.163	**< 0.001**	0.769	**< 0.001**	0.460	0.346
	12	15.1 ± 0.51	15.2 ± 0.68	14.8 ± 0.75	15.4 ± 0.36							
	24	16.1 ± 0.51	16.5 ± 0.49	16.9 ± 0.58	16.8 ± 0.32							
**Tibia mass (Of tibia length; mg/mm)**	6	2.807 ± 0.247	2.894 ± 0.342	2.581 ± 0.427	2.544 ± 0.273	**0.002**	0.064	**< 0.001**	0.069	0.292	0.183	0.828
	12	3.110 ± 0.247	3.125 ± 0.301	2.980 ± 0.356	3.035 ± 0.477							
	24	3.549 ± 0.248	3.819 ± 0.315	3.470 ± 0.117	3.695 ± 0.265							
**Femur mass (Of femur length; mg/mm)**	6	4.092 ± 0.347	4.027 ± 0.392	3.566 ± 0.528	3.480 ± 0.256	< **0.001**	0.619	**< 0.001**	0.767	0.073	0.447	0.994
	12	4.546 ± 0.278	4.611 ± 0.326	4.376 ± 0.572	4.386 ± 0.576							
	24	5.566 ± 0.521	5.731 ± 0.349	5.393 ± 0.234	5.511 ± 0.429							

Table S14. Physiological parameters of isolated tibialis anterior muscles from male wildtype and *mdx* offspring born to non-stressed or stressed *mdx*-heterozygous mothers at 6, 12 and 24 weeks of age. Values are mean ± SD. CSA = Cross-sectional area, ECC = eccentric contractions. Three different stress paradigms were employed including a non-stressed condition, and a stressed condition which combined data from the 30-second scruff restraint and 30-minute tube restraint paradigms.

**Parameter**	**Age (weeks)**	**Wildtype**		***mdx***		**Genotype** (p value)	**Paradigm** (p value)	**Time** (p value)	**Genotype x Paradigm** (p value)	**Genotype x Time** (p value)	**Paradigm x Time** (p value)	**Genotype x Paradigm x Time** (p value)
		Non-stressed	Stressed	Non-stressed	Stressed							
**CSA (cm^2)**	6	0.05 ± 0.01	0.05 ± 0.01	0.06 ± 0.01	0.06 ± 0.01	**< 0.001**	0.245	**< 0.001**	0.749	**< 0.001**	0.247	0.710
	12	0.06 ± 0.01	0.06 ± 0.00	0.09 ± 0.01	0.09 ± 0.01							
	24	0.07 ± 0.01	0.07 ± 0.01	0.10 ± 0.01	0.11 ± 0.01							
**Isometric twitch force (mN)**	6	233 ± 41.4	225 ± 53.4	203 ± 38.5	189 ± 53.1	0.465	0.524	**< 0.001**	0.582	**0.029**	0.881	0.547
	12	299 ± 62.5	277 ± 60.4	299 ± 71.0	323 ± 70.1							
	24	323 ± 113	314 ± 60.3	359 ± 84.8	346 ± 48.2							
**Specific isometric twitch force (N/cm^2)**	6	4.76 ± 0.59	4.65 ± 1.05	3.48 ± 0.67	3.23 ± 0.77	**< 0.001**	0.236	0.127	0.391	0.676	0.835	0.538
	12	5.44 ± 1.14	4.90 ± 1.20	3.50 ± 0.69	3.79 ± 0.90							
	24	5.05 ± 2.03	4.65 ± 0.90	3.33 ± 0.84	3.02 ± 0.46							
**Isometric tetanic force (mN)**	6	1028 ± 124	654 ± 111	815 ± 120	866 ± 225	0.928	0.878	**< 0.001**	0.271	**0.003**	0.790	0.829
	12	1303 ± 140	1317 ± 172	1355 ± 227	1412 ± 259							
	24	1332 ± 182	1300 ± 185	1396 ± 194	1407 ± 227							
**Specific isometric tetanic force (N/cm^2)**	6	21.2 ± 2.86	19.8 ± 2.27	14.0 ± 2.22	15.04 ± 2.61	**< 0.001**	0.370	**< 0.001**	0.132	0.396	0.642	0.713
	12	23.8 ± 2.89	23.2 ± 3.10	15.9 ± 2.14	16.4 ± 2.74							
	24	20.7 ± 3.96	19.3 ± 2.74	13.0 ± 2.45	12.3 ±2.24							
**Twitch : Tetanic**	6	0.23 ± 0.04	0.23 ± 0.04	0.25 ± 0.04	0.22 ± 0.03	0.348	0.469	**0.041**	0.577	**0.**894	0.857	0.261
	12	0.23 ± 0.05	0.21 ± 0.04	0.22 ± 0.05	0.23 ± 0.04							
	24	0.24 ± 0.06	0.25 ± 0.07	0.26 ± 0.07	0.25 ± 0.05							

**Isometric twitch time to peak (ms)**	6	59 ± 2.17	62.6 ± 22.5	55.1 ± 18.6	88.5 ± 62.1	0.180	0.964	0.722	0.664	0.799	0.067	0.310
	12	72 ± 36.6	63.8 ± 19.1	78.1 ± 50.8	62.5 ± 14.1							
	24	67.0 ± 34.8	65.3 ± 35.6	82.4 ± 44.5	71.3 ± 15.2							
**Isometric twitch 1/2 relaxation time (ms)**	6	20.7 ± 5.92	21.2 ± 6.04	22.9 ± 4.26	21.4 v 6.46	0.590	0.668	< **0.001**	0.698	0.210	0.938	0.595
	12	16.0 ± 1.51	15.5 ± 1.81	14.3 ± 1.06	14.8 ± 1.80							
	24	14.8 ± 1.21	14.7 ± 1.30	13.9 ± 1.52	13.5 ± 0.60							
**Isometric tetanic rate of contraction (N/s)**	6	24.6 ± 5.42	23.6 ± 5.33	19.9 ± 5.72	20.8 ± 6.50	0.576	0.670	< **0.001**	0.589	0.063	0.429	0.915
	12	34.8 ± 7.49	36.1 ± 8.69	38.0 ± 12.7	39.2 ± 10.1							
	24	40.6 ± 7.29	36.3 ± 7.47	42.1 ± 6.26	40.4 ± 9.21							
**Isometric tetanic rate of relaxation (N/s)**	6	22.1 ± 7.16	19.6 ± 6.18	19.4 ± 11.0	19.7 ± 8.18	**0.010**	0.894	< **0.001**	0.795	0.068	0.779	0.768
	12	30.8 ± 10.1	33.9 ± 12.2	40.8 ± 11.7	40.9 ± 12.6							
	24	29.1 ± 6.41	29.3 ± 11.3	37.9 ± 15.3	35.2 ± 14.7							
**Maximal eccentric force (mN)**	6	1991 ± 199	2037 ± 203	1975 ± 202	1911 ± 2536	0.974	0.554	< **0.001**	0.924	0.396	0.258	0.384
	12	2407 ± 256	2462 ± 223	2279 ± 303	2536 ± 370							
	24	2287 ± 393	2297 ± 379	2452 ± 338	2339 ± 487							
**Specific eccentric force (N/cm2)**	6	40.9 ± 2.88	42.3 ± 4.72	33.8 ± 6.16	33.9 ± 6.06	**< 0.001**	0.956	< **0.001**	0.837	**0.001**	0.315	0.512
	12	44.0 ± 5.86	43.4 ± 4.25	26.9 ± 3.17	22.5 ± 4.53							
	24	35.4 ± 7.66	34.0 ± 5.37	22.8 ± 4.60	20.5 ± 4.50							
**Tetanic force (% of initial post ECC)**	6	62.6 ± 16.5	64.3 ± 12.5	39.3 ± 10.7	42.7 ± 8.93	**< 0.001**	0.330	0.082	0.393	**0.041**	0.808	0.206
	12	57.2 ± 8.50	71.2 ± 11.4	31.2 ± 10.2	26.8 ± 13.8							
	24	72.9 ± 14.0	71.9 ± 18.0	34.2 ± 19.7	36.1 ± 19.1							
**Isometric tetanic rate of contraction (% of initial post ECC)**	6	55.0 ± 16.1	56.7 ± 17.4	43.1 ± 14.6	44.3 ± 11.9	**< 0.001**	0.451	< **0.001**	0.427	0.922	0.112	0.669
	12	55.2 ± 11.4	64.3 ± 13.7	36.3 ± 12.2	30.9 ± 15.8							
	24	73.6 ± 17.5	78.8 ± 23.7	46.4 ± 27.4	50.2 ± 28.0							

**Isometric tetanic rate of relaxation (% of initial post ECC)**	6	76.8 ± 24.5	72.9 ± 10.5	46.8 ± 12.1	47.5 ± 8.33	**< 0.001**	0.966	0.331	0.710	**< 0.001**	0.866	0.442
	12	74.2 ± 9.83	85.3 ± 14.4	31.9 ± 8.80	26.7 ± 17.7							
	24	96.1 ± 23.8	93.4 ± 35.7	24.2 ± 20.0	25.1 ± 12.1							

Table S15. Bone microarchitectural parameters from male wildtype and *mdx* offspring born to non-stressed or stressed *mdx*-heterozygous mothers at 6, 12 and 24 weeks of age. Values are mean ± SD. Three different stress paradigms were employed including a non-stressed condition, and a stressed condition which combined data from the 30-second scruff restraint and 30-minute tube restraint paradigms.

Parameter	Age (weeks)	Wildtype		*mdx*		Genotype (*p* value)	Paradigm (*p* value)	Time (*p* value)	Genotype x Paradigm (*p* value)	Genotype x Time (*p* value)	Paradigm x Time (*p* value)	Genotype x Paradigm x Time (*p* value)
		Non-stressed	Stressed	Non-stressed	Stressed							
**Femur length (mm)**	6	11.2 ± 0.25	11.26 ± 0.34	10.8 ± 0.47	10.8 ± 0.34	0.569	0.098	**< 0.001**	0.950	**< 0.001**	0.402	0.997
	12	12.7 ± 0.16	12.7 ± 0.18	12.7 ± 0.34	12.73 ± 0.35							
	24	13.0 ± 0.15	13.2 ± 0.23	13.4 ± 0.21	13.5 ± 0.17							
**Trabecular volume (% of tissue volume)**	6	10.4 ± 3.24	10.1 ± 2.61	6.41 ± 2.24	4.82 ± 1.61	**< 0.001**	0.479	**< 0.001**	0.095	**< 0.001**	0.424	0.971
	12	7.13 ± 3.50	8.29 ± 2.02	5.05 ± 4.04	4.61 ± 1.86							
	24	4.03 ± 1.70	4.33 ± 1.64	4.24 ± 0.89	3.40 ± 1.20							
**Trabecular number (1/mm)**	6	1.67 ± 0.47	1.64 ± 0.37	1.12 ± 0.36	0.87 ± 0.26	**< 0.001**	0.395	**< 0.001**	0.099	0.499	**< 0.001**	0.953
	12	1.19 ± 0.57	1.35 ± 0.31	0.91 ± 0.66	0.82 ± 0.31							
	24	0.72 ± 0.31	0.74 ± 0.24	0.77 ± 0.17	0.63 ± 0.19							
**Trabecular thickness (µm)**	6	62.1 ± 3.91	61.6 ± 3.11	56.6 ± 3.33	55.2 ± 2.73	**< 0.001**	0.775	**0.001**	0.228	0.124	0.138	0.620
	12	58.7 ± 6.08	61.2 ± 3.21	53.2 ± 4.93	55.0 ± 4.37							
	24	56.6 ± 4.94	57.8 ± 3.85	55.5 ± 2.06	53.1 ± 4.60							
**Trabecular separation (µm)**	6	377 ± 91.2	368 ± 57.7	428 ± 98.0	518 ± 110	**< 0.001**	0.083	**< 0.001**	**0.040**	**0.001**	0.354	0.246
	12	336 ± 70.9	326 ± 43.8	358 ±76.0	371 ± 48.5							
	24	335 ± 50.7	343 ± 31.4	325 ± 22.9	350 ± 30.7							
**Cortical thickness (µm)**	6	183 ± 12.2	182 ± 15.4	164 ± 17.1	162 ± 11.5	**< 0.001**	0.893	**< 0.001**	0.760	**< 0.001**	0.767	0.748
	12	220 ± 11.0	217 ± 13.0	215 ± 16.6	216 ± 12.3							
	24	222 ± 7.41	226 ± 8.25	218 ± 4.51	217 ± 8.04							
**Marrow area (mm^2)**	6	1.53 ± 0.16	1.51 ± 0.11	1.37 ± 0.21	1.36 ± 0.10	**0.007**	0.069	**0.009**	0.697	0.127	0.217	0.532
	12	1.43 ± 0.11	1.45 ± 0.18	1.33 ± 0.21	1.45 ± 0.19							
	24	1.47 ± 0.15	1.60 ± 0.18	1.46 ± 0.14	1.54 ± 0.19							

**Cortical area (mm^2)**	6	0.84 ± 0.09	0.83 ± 0.10	0.71 ± 0.13	0.69 ± 0.08	**< 0.001**	0.277	**< 0.001**	0.636	**0.008**	0.379	0.850
	12	1.02 ± 0.09	1.04 ± 0.06	0.99 ± 0.13	1.01 ± 0.11							
	24	1.06 ± 0.08	1.11 ± 0.06	1.04 ± 0.04	1.05 ± 0.08							
**Periosteal perimeter (mm)**	6	5.94 ± 0.28	5.92 ± 0.26	5.52 ± 0.48	5.45 ± 0.22	**< 0.001**	0.100	**< 0.001**	0.978	**0.005**	0.196	0.548
	12	5.99 ± 0.24	6.06 ± 0.28	5.81 ± 0.44	6.03 ± 0.35							
	24	6.11 ± 0.29	6.35 ± 0.28	6.13 ± 0.21	6.24 ± 0.33							
**Endocortical perimeter (mm)**	6	4.86 ± 0.28	4.84 ± 0.25	4.53 ± 0.38	4.57 ± 0.19	**0.022**	**0.021**	**< 0.001**	0.820	0.071	0.314	0.728
	12	4.75 ± 0.22	4.89 ± 0.33	4.66 ± 0.43	4.82 ± 0.34							
	24	4.84 ± 0.26	5.12 ± 0.37	4.90 ± 0.24	5.04 ± 0.34							
**Mean polar moment of inertia (mm^4)**	6	0.54 ± 0.10	0.53 ± 0.10	0.41 ± 0.13	0.38 ± 0.07	**< 0.001**	0.088	**< 0.001**	0.944	0.058	0.169	0.584
	12	0.65 ± 0.10	0.67 ± 0.10	0.58 ± 0.16	0.66 ± 0.13							
	24	0.70 ± 0.12	0.79 ± 0.11	0.69 ± 0.08	0.73 ± 0.13							

Table S16. Bone mechanical parameters from male wildtype and *mdx* offspring born to non-stressed or stressed *mdx*-heterozygous mothers at 24 weeks of age. Values are mean ± SD. Three different stress paradigms were employed including a non-stressed condition, and a stressed condition which combined data from the 30-second scruff restraint and 30-minute tube restraint paradigms. CC, cranio-caudal; ML, medial–latera.

Parameter	Age (weeks)	Wildtype		*mdx*		Genotype (*p* value)	Paradigm (*p* value)	Genotype x Paradigm (*p* value)
		Non-stressed	Stressed	Non-stressed	Stressed			
**Diameter ML (mm)**	24	2.15 ± 0.09	2.21 ± 0.10	2.21 ± 0.09	2.26 ± 0.11	0.096	0.066	0.860
**Diameter CC (mm)**	24	1.59 ± 0.08	1.65 ± 0.06	1.55 ± 0.06	1.57 ± 0.07	**0.006**	**0.040**	0.537
**Ultimate strain (%)**	24	5.20 ± 1.23	4.96 ± 1.06	5.52 ± 1.54	5.44 ± 1.39	0.297	0.678	0.830
**Yield displacement (mm)**	24	0.21 ± 0.04	0.20 ± 0.04	0.27 ± 0.07	0.25 ± 0.04	**< 0.001**	0.327	0.535
**Yield strain (%)**	24	3.15 ± 0.60	3.17 ± 0.58	3.91 ± 1.02	3.57 ± 0.56	**0.006**	0.427	0.394
**Post yield strain (%)**	24	4.13 ± 3.72	4.61 ± 3.74	5.43 ± 6.24	4.56 ± 5.38	0.662	0.892	0.636
**Failure deformation (mm)**	24	0.48 ± 0.22	0.51 ± 0.24	0.65 ± 0.45	0.54 ± 0.38	0.299	0.671	0.496
**Failure strain (%)**	24	7.28 ± 3.71	7.78 ± 3.49	9.34 ± 6.38	8.12 ± 5.64	0.410	0.802	0.554
**Failure energy (J)**	24	0.006 ± 0.004	0.007 ± 0.004	0.008 ± 0.006	0.006 ± 0.005	0.747	0.937	0.437
**Toughness (J/mm3)**	24	0.0009 ± 0.0005	0.001 ± 0.0007	0.001 ± 0.0009	0.0008 ± 0.0006	0.884	0.652	0.283
**Yield force (N)**	24	14.3 ± 3.79	16.0 ± 3.54	15.2 ± 2.36	15.0 ± 2.60	0.931	0.433	0.305
**Yield stress (MPa)**	24	13.8 ± 4.30	14.1 ± 4.28	14.9 ± 2.09	13.6 ± 3.40	0.802	0.664	0.470
**Stiffness (N/mm)**	24	81.3 ± 13.7	96.4 ± 11.5	76.5 ± 17.9	77.0 ± 17.5	**0.009**	0.087	0.108
**Post yield displacement (mm)**	24	0.27 ± 0.23	0.30 ± 0.25	0.38 ± 0.44	0.30 ± 0.35	0.568	0.804	0.576
**Ultimate force (N)**	24	18.3 ± 2.18	20.02 ± 2.75	18.0 ± 3.51	18.1 ± 3.10	0.224	0.289	0.325
**Elastic modulus (MPa)**	24	512 ± 111	548 ± 128	523 ± 166	476 ± 151	0.470	0.887	0.325
**Ultimate stress (MPa)**	24	17.8 ± 4.15	17.5 ± 3.98	17.7 ± 3.66	16.2 ± 2.82	0.531	0.428	0.582
**Ultimate displacement (mm)**	24	0.34 ± 0.07	0.32 ± 0.06	0.38 ± 0.10	0.37 ± 0.09	0.062	0.536	0.924
**Failure force (N)**	24	16.5 ± 2.39	17.6 ± 3.89	14.7 ± 5.56	14.9 ± 5.10	0.095	0.630	0.687
**Failure stress (MPa)**	24	16.1 ± 4.49	15.2 ± 3.53	14.1 ± 4.40	13.0 ± 4.60	0.089	0.397	0.924
